# A multidimensional coding architecture of the vagal interoceptive system

**DOI:** 10.1038/s41586-022-04515-5

**Published:** 2022-03-16

**Authors:** Qiancheng Zhao, Chuyue D. Yu, Rui Wang, Qian J. Xu, Rafael Dai Pra, Le Zhang, Rui B. Chang

**Affiliations:** 1 Department of Neuroscience, Yale University School of Medicine, New Haven, CT, USA.; 2 Department of Cellular and Molecular Physiology, Yale University School of Medicine, New Haven, CT, USA.; 3 Interdepartmental Neuroscience Program, Yale University School of Medicine, New Haven, CT, USA.; 4 Department of Neurology, Yale University School of Medicine, New Haven, CT, USA.

## Abstract

Interoception, the ability to timely and precisely sense changes inside the body, is critical for survival^1–4^. Vagal sensory neurons (VSNs) form an important body-to-brain connection, navigating visceral organs along the rostral–caudal axis of the body and crossing the surface–lumen axis of organs into appropriate tissue layers^5,6^. The brain can discriminate numerous body signals through VSNs, but the underlying coding strategy remains poorly understood. Here we show that VSNs code visceral organ, tissue layer and stimulus modality—three key features of an interoceptive signal—in different dimensions. Large-scale single-cell profiling of VSNs from seven major organs in mice using multiplexed projection barcodes reveals a ‘visceral organ’ dimension composed of differentially expressed gene modules that code organs along the body’s rostral–caudal axis. We discover another ‘tissue layer’ dimension with gene modules that code the locations of VSN endings along the surface–lumen axis of organs. Using calcium-imaging-guided spatial transcriptomics, we show that VSNs are organized into functional units to sense similar stimuli across organs and tissue layers; this constitutes a third ‘stimulus modality’ dimension. The three independent feature-coding dimensions together specify many parallel VSN pathways in a combinatorial manner and facilitate the complex projection of VSNs in the brainstem. Our study highlights a multidimensional coding architecture of the mammalian vagal interoceptive system for effective signal communication.

Sensing the body’s internal state is a critical life-ensuring process that maintains physiological homeostasis, provides motivational drivers and shapes our thoughts and emotions^1–4^. As a key body–brain axis in interoception, VSNs in the nodose and jugular ganglia transmit numerous signals from visceral organs in the respiratory, cardiovascular, gastrointestinal, endocrine and immune systems into the brainstem^5,6^. Signals communicated through VSNs are precisely discriminated in the brain for highly specific responses^5^, yet how this is achieved remains unclear. Extensive data show that VSNs are highly heterogeneous in multiple characteristics, including developmental origins, electrical properties, response patterns, molecular identities, terminal morphologies, sensory mechanisms, anatomical connections and physiological roles^5–19^. However, despite the accumulation of a large amount of data over the past seven decades describing the complexity of VSN characteristics, little is known about how such heterogeneity and diversity facilitates interoceptive coding at a systems level.

The physiological role of an interoceptive signal can be specified by three important features: visceral organ; tissue layer; and stimulus modality. Stimuli that differ in these features represent different body changes. For example, stretching the arterial wall implicates an increase in blood pressure, whereas stretching the stomach wall signals food ingestion1^5,20^. Similarly, the same bioactive signal (such as serotonin) released from different tissue layers within the intestine communicates different organ information to the brain^21^. We reasoned that a faithful coding of these features in VSNs is necessary for accurate signal discrimination in the brain. Here we developed several techniques to determine whether and how these essential features of interoceptive signals are coded in VSNs.

## ‘Visceral organ’ coding in VSNs

**Extended Data Fig. 1 |:**
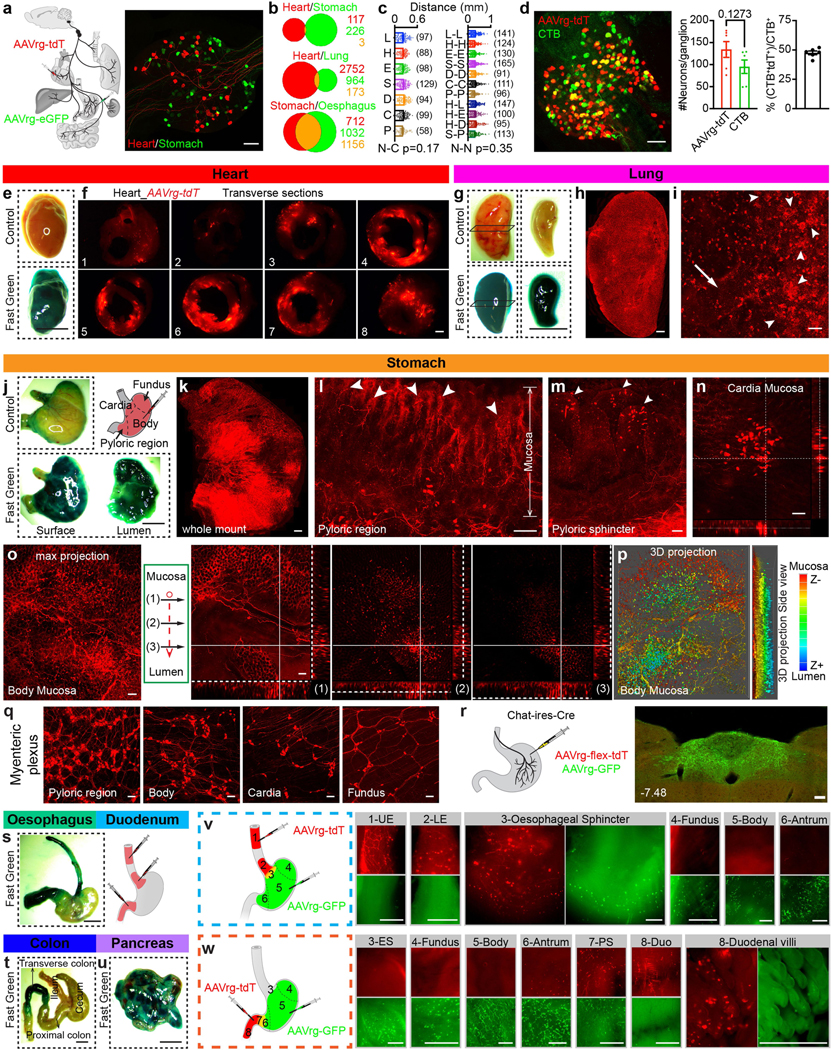
Developing and applying Projection-seq in VSNs. **a**, Retrograde labelling of VSNs in vagal ganglia from the heart (AAVrg-tdTomato, red) and the stomach (AAVrg-GFP, green). **b**, The numbers of VSNs singly (red or green) or dual (orange) labelled from indicated organs. n = 3–4 mice. **c**, Distance from a random neuron labelled from indicated organs to the centre of the ganglion (top) or between two neurons labelled from the same or two different organs (bottom). mean ± SEM, one-way ANOVA. L- lung; H- heart; S- stomach; E- oesophagus; D- duodenum; C- colon; P- pancreas. **d**, A representative image (left) and quantification (right, mean ± SEM, n = 6) of VSNs retrogradely labelled from the stomach using AAVrg-tdTomato (red) and Alexa Fluor™ 647 conjugated CTb (green), showing that VSNs are labelled by AAVrgs and CTb with comparable efficiencies. **e**, Mouse hearts without (top) and with (bottom) Fast Green FCF injection. **f**, A series of transverse heart sections (1 mm thickness) with heart injection of AAVrg-tdTomato, showing that AAVrg can cover all tissue layers of most ventricles. **g**, Whole-mount view (left) and transverse-section view (right, as indicated by the parallelogram in the left) of mouse lung lobes without (top) and with (bottom) lung injection of Fast Green FCF through a tracheal cannula. **h**, **i**, Whole-mount (**h**) and zoom-in (**i**) view of a lung lobe with lung injection of AAVrg-tdTomato, showing that AAVrg can cover alveoli cells in most regions of the lung. Arrow, bronchi; arrowheads, infected alveoli cells. **j**, Mouse stomachs without (top) and with (surface view, left bottom; lumen view, right bottom) Fast Green FCF injection. Cartoon image (top right) shows injected stomach regions. **k**, Whole-mount view (max projection of stacked images) of a stomach with AAVrg-tdTomato injection, showing that AAVrg can cover most subregions of the stomach. **l**, **m**, transverse view around the pyloric region (**l**) and the pyloric sphincter (**m**) of a stomach with AAVrg-tdTomato injection, showing infection of the mucosal lining (arrowhead). **n**–**p**, lumen view of cardiac mucosa (**n**) and body mucosa (**o**, max projection (left) and at three different levels towards the lumen (right); **p**, 3D projection, colour-coded) of a stomach with AAVrg-tdTomato injection, showing infection of the mucosal lining. **q**, Infected enteric neurons in indicated subregions of a stomach with AAVrg-tdTomato injection. **r**, stomach injection with AAVrg-FLEX-tdTomato and AAVrg-GFP in Chat-ires-Cre mice (left) extensive labelled VSN central projections (green) but not DMV neurons (red). Organ illustration was adapted from BioRender.com. **s**–**u**, Mouse oesophagus (**s**), duodenum (**s**, cartoon image on the right showing injection areas), transverse colon (**t**) and pancreas (**u**) with corresponding organ injection of Fast Green FCF. **v**, Oesophagus (AAVrg-tdTomato) and stomach (AAVrg-GFP) co-injection, showing the distribution of infected tdTomato^+^ and GFP^+^ cells along subregions of the gastrointestinal tract. Oesophagus cells are heavily infected by tdTomato and stomach cells by GFP. Co-infection was only observed in region 3 around the oesophageal sphincter. UE, upper oesophagus; LE, lower oesophagus. **w**, Duodenum (AAVrg-tdTomato) and stomach (AAVrg-GFP) co-injection, showing the distribution of infected tdTomato^+^ and GFP^+^ cells along subregions of the gastrointestinal tract. Duodenum cells, including mucosal epithelial cells in the villi, are heavily infected by tdTomato and stomach cells by GFP. Co-infection was only observed in region 6 and 7 around the antrum and pyloric sphincter. Scale bars: 5 mm (**e**, **g**, **j**, **s**, **t**, **u**), 1 mm (**f**, **h**, **k**, **v**, **w**), 100 μm (others). Cartoon illustrations in **a**, **j**, **s**–**w** adapted with permission from ref. ^11^.

A gross topographic organization of visceral organs exists in paraver-tebral ganglia along the sympathetic chain and dorsal root ganglia (DRG) along the spinal cord^22,23^. Whether a viscerotopic map exists in the nodose ganglion, where most visceral-organ-innervating VSNs are located^6^, is a subject of debate^12,24–27^. Using retrograde adeno-associated virus (AAVrg), we show that VSNs labelled from various visceral organs are largely non-overlapping (Extended Data Fig. 1a–d), which suggests that organ information is encoded within VSNs. However, their salt-and-pepper distribution patterns indicate that a viscerotopic map is missing in the nodose ganglion.

**Extended Data Fig. 2 |:**
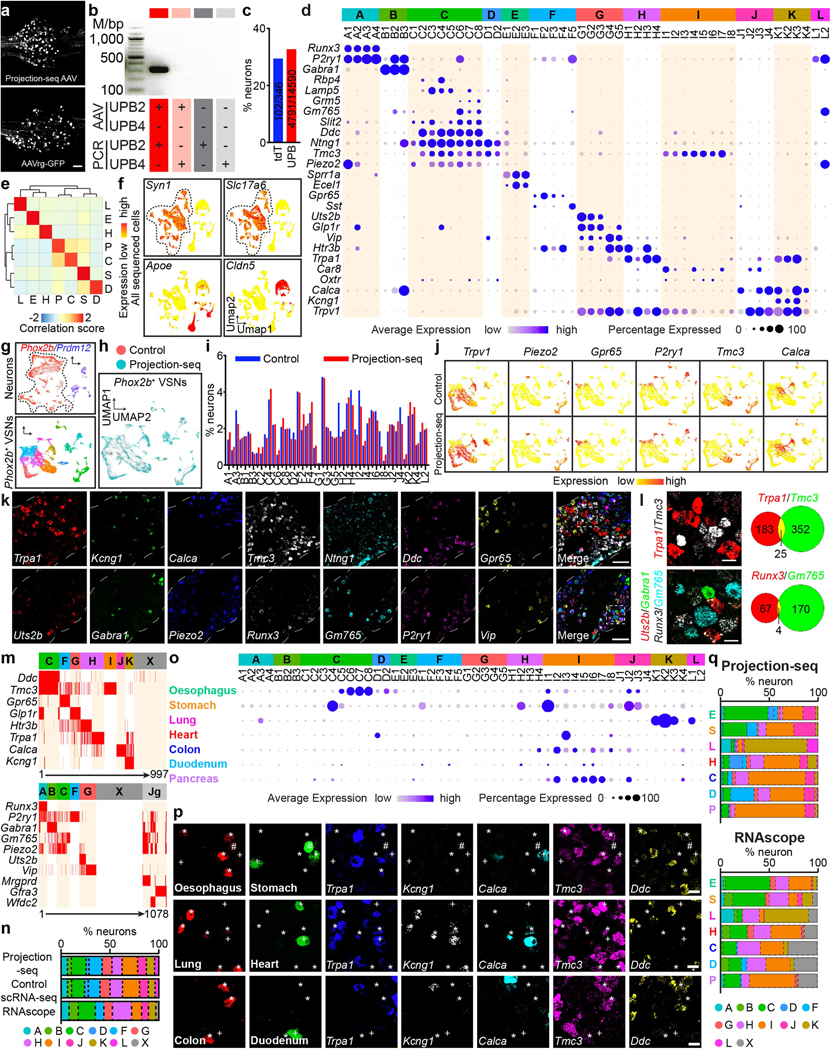
Projection-seq analysis faithfully reveals the molecular architecture and organ projection of VSNs. **a**, VSNs retrogradely labelled from the stomach using Projection-seq AAV (UPB-stomach, top) or AAVrg-GFP (bottom). **b**, RT-PCR analysis of vagal ganglia cDNA from mice with stomach injection of Projection-seq AAV-UPB2 (red) or control (grey) mice using primers that recognize UPB2 (dark colour) or UPB4 (light colour) sequences. For gel source data, see Supplementary Fig. 1. **c**, Percentage of tdTomato^+^ neurons after acute VSN dissociation (blue) and UPB marked neurons after Projection-seq analysis (red). **d**, Dot plot of expression of indicated marker genes in 12 VSN subpopulations (A-L)/52 VSN clusters (A1-L2). **e**, Correlation scores for VSNs labelled with UPBs from indicated visceral organs. **f**, UMAP plots of 31,182 cells from control scRNA-seq, coloured by expression of indicated genes. Dashed circle indicates *Syn1*^+^/*Slc17a6*^+^ neuronal clusters. **g**, (top) UMAP plot of 16,476 neurons from neuronal clusters indicated in (**f**), coloured by expression of indicated genes, showing the two developmental origins of VSNs. *Prdm12* (blue) labels neural crest derived VSNs in the jugular ganglia. *Phox2b*^+^ placode derived clusters (red, dashed circle) containing 13,210 VSNs in the nodose ganglia from control scRNA-seq data are re-clustered and plotted on the UMAP plot (bottom), coloured by VSN subpopulations. **h**, UMAP plot of 27,800 *Phox2b*^+^ placode-derived VSNs from integrated Projection-seq (cyan) and control scRNA-seq (red) data. **i**, Percentage of neurons in 49 VSN clusters from control scRNA-seq (blue) and Projection-seq (red) datasets. E-VSNs were excluded. **j**, UMAP plots of VSNs colour by expression of indicated marker genes from control scRNA-seq (top) and Projection-seq (bottom) data. **k**, RNAscope HiPlex Assay in the nodose/ jugular ganglia for indicated marker genes identified from Projection-seq. **l**, Zoom-in images from the dashed regions in (**k**). The numbers of VSNs expressing one gene (red or green) or both genes (yellow) were counted. Consistent with Projection-seq data as shown in (**d**), *Trpa1* and *Tmc3* were largely expressed in non-overlapping VSNs, whereas *Ut2sb*, *Runx3*, *Gabra1*, and *Gm765* each labelled a distinct VSN subset. **m**, VSN subpopulations determined from RNAscope HiPlex Assays using indicated genes identified by Projection-seq. One neuron per column as indicated in the bottom. X, unlabelled; Jg, jugular neurons. **n**, Cumulative percentage of neurons in 11 VSN subpopulations revealed by Projection-seq, control scRNA-seq, or RNAscope. X, unlabelled. **o**, Dot plot of expression of UPBs representing indicated organs in 12 VSN subpopulations (A-L)/52 clusters (A1-L2). **p**, RNAscope HiPlex Assays for indicated genes. VSNs were retrogradely labelled from indicated organs with AAVrgs and visualized using corresponding RNAscope probes. *, VSNs from oesophagus, lung, or colon. +, VSNs from stomach, heart, or duodenum. #, double-labelled VSNs. **q**, Cumulative percentage of neurons in 11 VSN subpopulations expressing organ UPBs from Projection-seq analysis (top) and retrogradely labelled from indicated organs from RNAscope analysis (bottom). X, unlabelled. Scale bars: 100 μm (**a**, **k**), 20 μm (**l**, **p**).

**Fig. 1 |:**
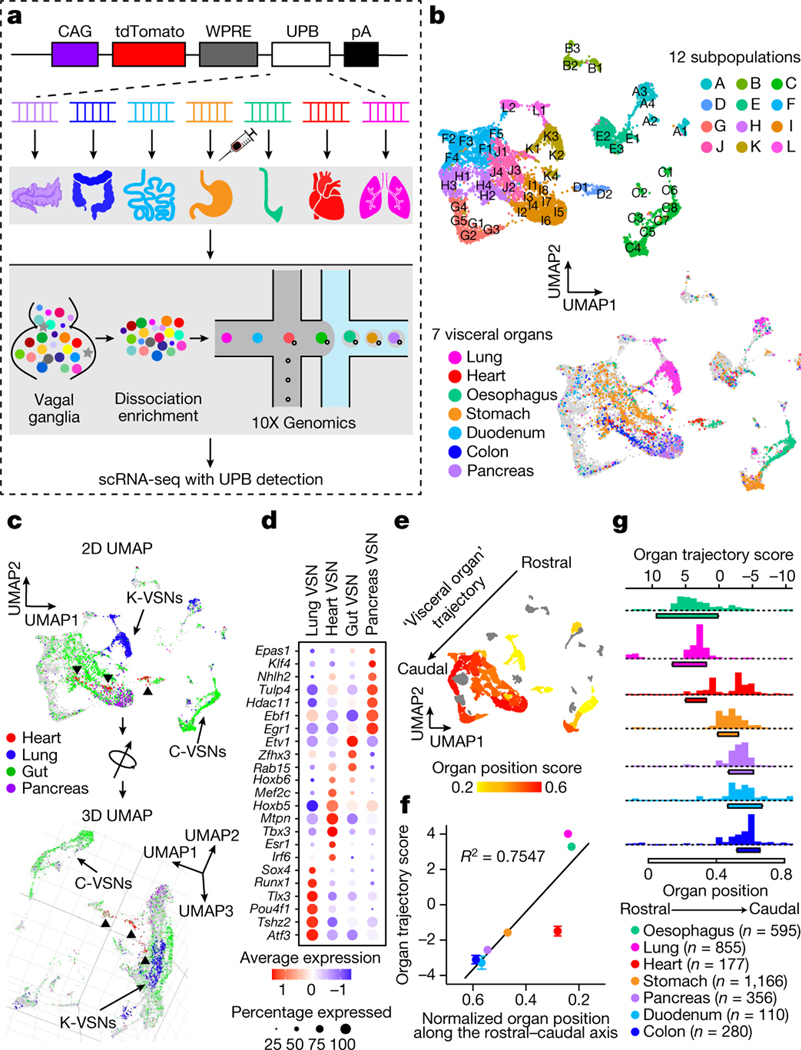
‘Visceral organ’ coding in VSNs. **a**, Schematic illustration of Projection-seq analysis of VSNs innervating the lung, heart, oesophagus, stomach, duodenum, transverse colon and pancreas. Organ illustrations were adapted from BioRender.com. **b**, UMAP plot from Projection-seq of 14,590 *Phox2b*^*+*^ VSNs (30 mice divided into 4 samples) showing 52 clusters (A1–L2) in 12 VSN subpopulations (A–L) (top) or VSNs expressing UPBs representing 7 visceral organs (colour-coded) (bottom). **c**, Two-dimensional (2D) (top) and three-dimensional (3D) (bottom) UMAP plots of VSNs innervating different physiological systems. E-VSNs were excluded. The three heart VSN groups (red, arrowheads) are clustered together away from other gut VSNs (green) in the 3D UMAP plot. **d**, Dot plot showing transcription factors that are differentially expressed in lung, heart, gut and pancreas VSNs. **e**, UMAP plot of VSN clusters, coloured by target preference (weighted organ position score), showing a ‘visceral organ’ trajectory (arrow) coding visceral organs along the body’s rostral–caudal axis. **f**, Correlation between the normalized position of the indicated organs along the body’s rostral–caudal axis (mean; *n* = 4) and the position of VSNs expressing indicated organ UPBs along the ‘visceral organ’ trajectory (organ trajectory score; mean ± s.e.m.; *n* as indicated). Linear regression *R*^2^ = 0.7547. **g**, Histograms showing the distributions of UPB-labelled VSNs (colour-coded) along the identified ‘visceral organ’ trajectory. The bars underneath indicate normalized organ positions along the body’s rostral– caudal axis (beginning–end; mean ± s.e.m.; *n* = 4).

We therefore hypothesize that visceral organs are coded by specific genes in VSNs. We developed a single-cell sequencing approach named ‘Projection-seq’ for unbiased, high-throughput genetic and anatomical dissection of complex neural circuits (Fig. 1a). Engineered from AAVrgs, Projection-seq AAVs encode a series of unique projection barcodes (UPBs) composed of exogenous nucleotide sequences, thus enabling large-scale multiplex sequencing with neuronal projection information. We injected Projection-seq AAVs with different UPBs into seven major visceral organs of the same mice, including the lung, heart, stomach, oesophagus, duodenum, pancreas and transverse colon (Fig. 1a). AAVrgs cover most subregions in all examined organs and specificity is ensured by no labelling of vagal motor neurons (Extended Data Fig. 1e–w). VSNs that project to adjoining regions between two organs, such as the oesophageal and pyloric sphincters, can be accurately identified with UPBs from both organs as AAVrg slightly diffuses to adjacent areas (Extended Data Fig. 1v, w). The infection efficiencies of Projection-seq AAVs were comparable to regular AAVrgs, and UPBs were successfully detected from retrogradely infected VSNs (Extended Data Fig. 2a–c).

**Extended Data Fig. 3 |:**
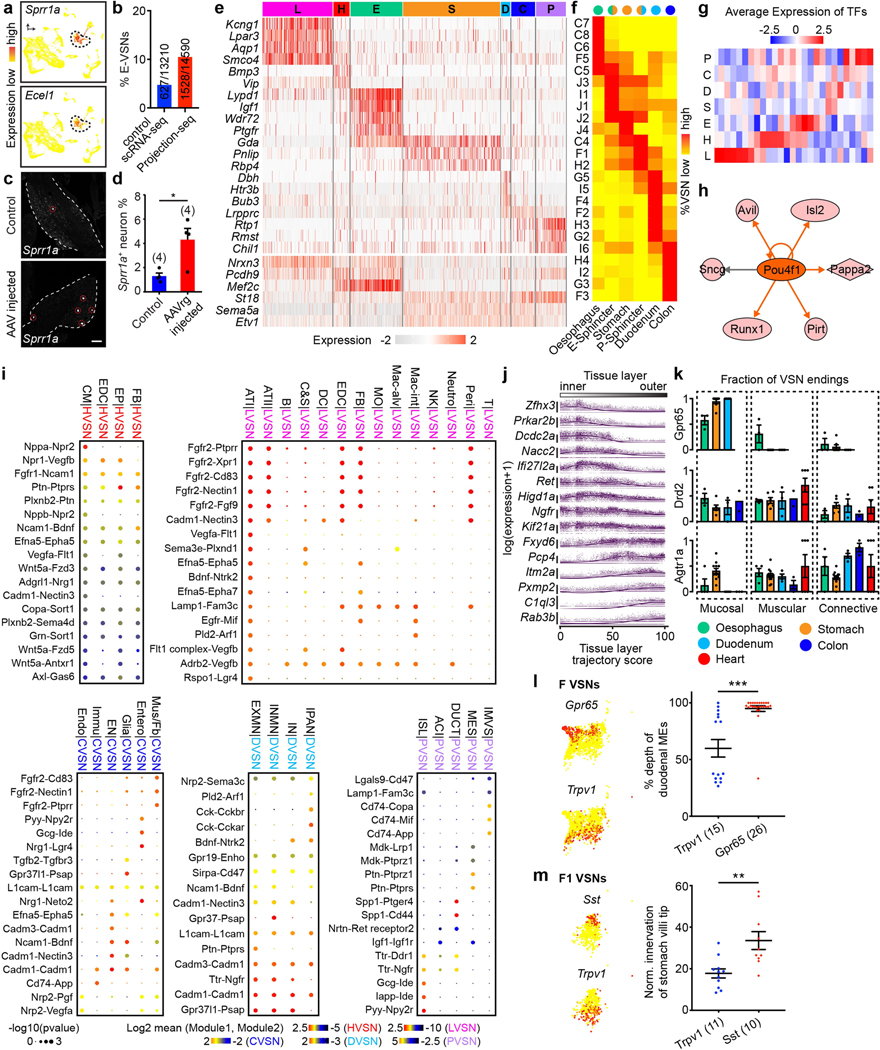
‘Visceral organ’ and ‘tissue layer’ coding in VSNs. **a**, UMAP plots of VSNs showing that *Sprr1a* (top) and *Ecel1* (bottom), genes upregulated in damaged sensory neurons, are selectively expressed in E-VSNs (dashed circles, control scRNA-seq data). **b**, Percentage of E-VSNs in label-free control scRNA-seq data (blue) and Projection-seq (red) data. **c**, Detection of *Sprr1a* using RNAscope in vagal ganglia from control (top) and AAVrg stomach injected (bottom) mice. Dashed lines indicate the shape of vagal ganglia. *Sprr1a*^+^ neurons are indicated in red dashed circle. Scale bar: 100 μm. **d**, Percentage of *Sprr1a*^+^ VSNs in control versus AAVrg stomach injected mice. mean ± SEM, n = 4. *p < 0.05, p = 0.0204, two-tailed *t*-test. **e**, Heat map of genes differentially expressed in VSNs innervating indicated organs. **f**, Percentage of VSNs marked by UPBs from different gut regions in indicated clusters, coloured per cluster. E (oesophageal)-Sphincter VSNs express both UPB-oesophagus and UPB-stomach. P (pyloric)-Sphincter VSNs express both UPB-stomach and UPB-duodenum. **g**, Differential expression of transcription factors in UPB-marked VSNs innervating indicated organs. From left: *Atf3*, *Terf1*, *Runx1*, *Sox4*, *Tshz2*, *Pou4f1*, *Cebpb*, *Irf6*, *Esr1*, *Tbx3*, *Id3*, *Hoxb5*, *Carhsp1*, *Hoxb6*, *Mef2c*, *Zfhx3*, *Tcf4*, *Egr1*, *Klf2*, *Klf5*, *Casz1*, *Etv1*, *Epas1*, *Nfkbia*, *Scrt2*, *St18*, *Scx*, *Klf4*, and *Nhih2*. **h**, IPA predicted regulatory network of *Pou4f1* in lung VSNs. Orange arrows, predicted activation; grey arrow, unknown. **i**, Dot plots of cell-cell signalling between organ-UPB labelled VSNs and various cell types in corresponding organs, predicted by CellPhoneDB. (top left), HVSN, heart-VSN; CM, cardiomyocyte; EDC, endothelial cell; EP, epicardial cell; FB, fibroblast. (top right), LVSN, lung-VSN; ATI, alveolar epithelial type I cell; ATII, alveolar epithelial type II cell; B, B cell; C&S, ciliated and secretory cell; DC, dendritic cell; EDC, endothelial cell; FB, fibroblast; MO, monocyte; Mac, macrophage; NK, natural kill cell; Neutro, neurophil; Peri, pericyte; T, T cell. (bottom left), CVSN, colon-VSN; Endo, endothelial cell; Immu, immune cell; EN, enteric neuron; Glia, glial cell; Entero, enteroendocrine cell; Mus/Fb, muscle cell and fibroblast. (bottom middle), DVSN, duodenum-VSN; EXMN, excitatory motor neuron; INMN, inhibitory motor neuron; IN, inter neuron; IPAN, intrinsic primary afferent neuron. (bottom right), PVSN, pancreas-VSN; ISL, islet cell; ACI, acinar cell; DUCT, duct cell; MES, mesenchymal cell; IMVS, immune and vascular cell. **j**, Scatterplots of expression measures vs tissue layer trajectory scores for indicated DEGs along the tissue layer trajectory. Bars: 0–3. **k**, Fraction of DEG^+^ VSN endings characterized in Gpr65^tdT^, Drd2^tdT^, and Agtr1a^tdT^ mice in indicated tissue layers of indicated organs. mean ± SEM, number of mice: Gpr65-Oesophagus (3), Gpr65-Stomach (7), Gpr65-Duodenum (4), Drd2Oesophagus (3), Drd2-Stomach (6), Drd2-Duodenum (3), Drd2-Colon (2), Drd2-Heart (6), Agtr1a-Oesophagus (4), Agtr1a-Stomach (12), Agtr1a-Duodenum (4), Agtr1a-Colon (3), Agtr1a-Heart (6). **l**, Expression of *Gpr65* and *Trpv1* in F-VSNs (left) and maximum depths of duodenal MEs, normalized to the thickness of the sample, in Trpv1^tdT^ and Gpr65^tdT^ mice (right). mean ± SEM, 15 endings from 3 mice for Trpv1, 26 endings from 4 mice for Gpr65. ***p < 0.001, p = 6.6 × 10^−6^, two-tailed *t*-test. **m**, Expression of *Sst* and *Trpv1* in F1-VSNs (left) and the number of stomach gland tips innervated in the antrum and corpus, normalized to the innervation area, in Trpv1^tdT^ and Sst^tdT^ mice (right). mean ± SEM, 11 endings from 5 mice for Trpv1, 10 endings from 5 mice for Sst, **p < 0.01, p = 0.0031, two-tailed *t*-test.

In total 14,590 *Phox2b^+^* placode-derived nodose VSNs^13,17,18^ (of 25,393 cells after quality filtering) were grouped into 12 subpopulations (A–L) and 52 clusters (A1–L2) on the basis of differentially expressed genes (DEGs) using Seurat and visualized using uniform manifold approximation and projection (UMAP) (Fig. 1b, Extended Data Fig. 2d). VSNs labelled from each of the seven organs were identified using corresponding UPBs with extremely high detection efficiency (Fig. 1b, Extended Data Fig. 2c). The distribution of UPBs recapitulated AAVrg coverage in the organs (Extended Data Fig. 2e). VSN clusters and patterns of gene expression were similar among control label-free single-cell RNA sequencing (scRNA-seq), RNAscope HiPlex in situ hybridization and Projection-seq data (Extended Data Fig. 2f–n), showing that viral infection did not alter gene expression in VSNs. The genetic identities of organ-UPB-marked VSNs were further validated using RNAscope (Extended Data Fig. 2o–q). VSNs that are damaged by cell dissociation and viral infection can be easily identified (VSN subpopulation E, referred as E-VSNs; same for other subpopulations) and removed from analysis (Extended Data Fig. 3a–d). Together, Projection-seq results faithfully represent VSN clusters, gene expression and neuronal projections.

Projection-seq analysis reveals a genetic segregation of VSNs that innervate organs in the respiratory (lung), cardiovascular (heart), gastrointestinal (oesophagus, stomach, duodenum and transverse colon) and exocrine–endocrine (pancreas) systems (Fig. 1c, Extended Data Fig. 3e). Gut VSN clusters exhibit unique preferences for specific gastrointestinal regions (Extended Data Fig. 3f). DEG analysis identified a set of transcription factors that may help define VSN identity for visceral organs (Fig. 1d, Extended Data Fig. 3g–h), many of which are critically involved in the development and differentiation of sensory neurons^28–32^. Of note, it seems that VSNs are primed for the functions of their target organs. For example, the stomach contains several anatomically and functionally distinct subregions: the proximal region similar to the oesophagus and the distal region similar to the intestine^33^. Accordingly, UPB-stomach^+^/UPB-oesophagus^+^ VSNs are genetically similar to oesophagus VSNs, whereas UPB-stomach^+^/UPB-duodenum^+^ VSNs are closer to duodenum VSNs (Extended Data Fig. 3f). Consistent with this notion, cell–cell interaction analyses suggest that VSNs use different ligand–receptor complexes to communicate with cells in different organ systems (Extended Data Fig. 3i). Together, our results reveal that VSNs use differential gene module expression to code visceral organs.

Notably, we further identified a genetic trajectory that represents visceral organs along the body’s rostral–caudal axis (Fig. 1e). The relative positions of VSNs along this trajectory (‘visceral organ’ trajectory score) on the UMAP plot had a strong linear relationship with the locations of their target organs along the body’s rostral–caudal axis (Fig. 1f, g). Anatomically, most vagal afferents travel together along a general path before branching off to their target organs, instead of making organ-specific projection pathways^6^. Our discovery of a genetic ‘visceral organ’ trajectory is in accordance with this long-standing observation, suggesting that VSNs might follow morphogen gradients to their target spots. Indeed, signalling gradients formed by secreted morphogens help to establish an anterior–posterior patterning^34^ and specify peripheral targets for vagal motor neurons^35^. Together, our data show that instead of a topographic organization, VSNs use a genetic trajectory to code visceral organs along the body’s rostral–caudal axis.

## ‘Tissue layer’ coding specifies VSN endings

**Fig. 2 |:**
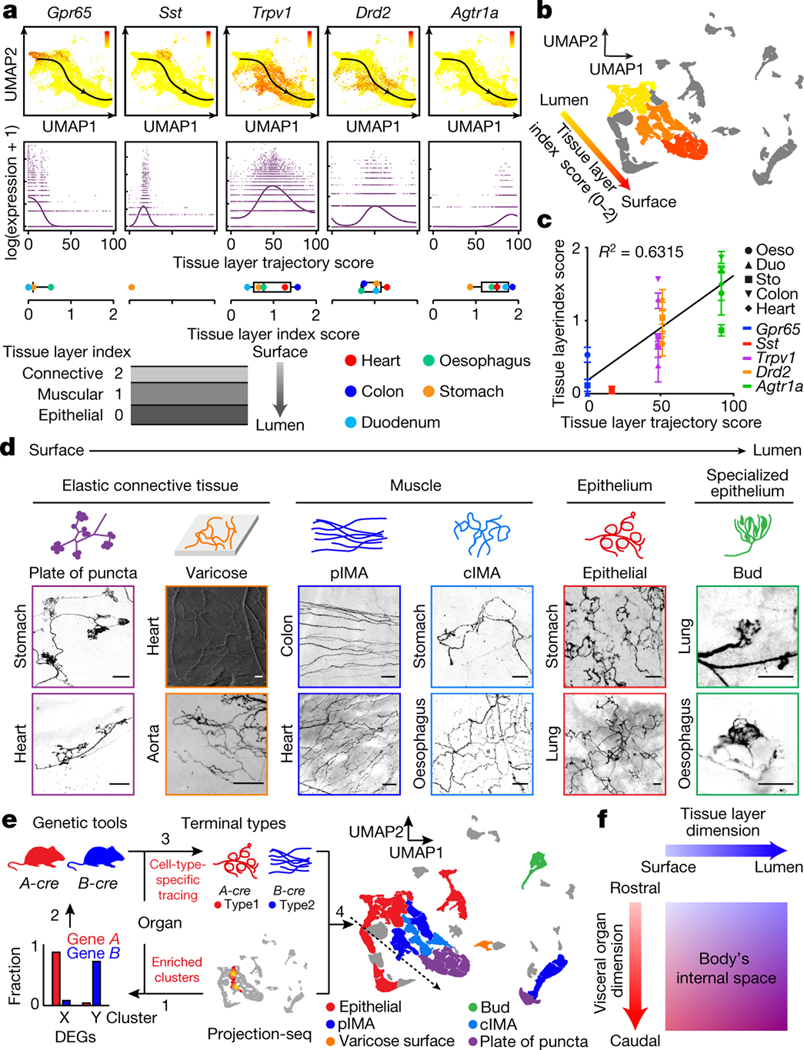
A ‘tissue layer’ dimension coding VSN ending locations and structures. **a**, UMAP plots of identified DEGs (top) and their expression measures (middle) along a ‘tissue layer’ trajectory. Bottom, DEG^+^ VSN ending locations, quantified as ‘tissue layer’ index score in corresponding DEG^tdT^ mice (mean; number of mice: *Gpr65*-oesophagus, 3; *Gpr65*-stomach, 7; *Gpr65*-duodenum, 4; *Sst*-stomach, 5; *Trpv1*-oesophagus, 3; *Trpv1*-stomach, 7; *Trpv1*-duodenum, 4; *Trpv1*-colon, 4; *Trpv1*-heart, 10; *Drd2*-oesophagus, 3; *Drd2*-stomach, 6; *Drd2*-duodenum, 3; *Drd2*-colon, 2; *Drd2*-heart, 6; *Agtr1a*-oesophagus, 4; *Agtr1a*-stomach, 12; *Agtr1a*-duodenum, 4; *Agtr1a*-colon, 3; *Agtr1a*-heart, 6). **b**, UMAP plot of VSN clusters, coloured by average tissue index determined in Gpr65^tdT^ (F1–F4 clusters; golden), Sst^tdT^ (F5 cluster; yellow), Drd2^tdT^ (J2–J4, H2, H4 and I1 clusters; orange), and Agtr1a^tdT^ (I2 and I4–6 clusters; orange-red) mice, showing a continuous trajectory coding tissue layers along the organ’s surface–lumen axis. **c**, Correlation between mean ‘tissue layer’ trajectory score of DEG^+^ VSNs and their ‘tissue layer’ index score in corresponding DEG^tdT^ mice (mean ± s.e.m.; *n* as in **a**). Linear regression *R*^2^ = 0.6315. **d**, VSN ending types characterized in Vglut2^tdT^ mice show stereotypical structures along various tissue layers across multiple visceral organs. Scale bars, 100 μm. **e**, Projection-seq-guided anterograde tracing (schematic illustration, left) reveals genetic identities of stereotypical VSN ending types illustrated on the UMAP plot (right). VSN clusters forming various VSN ending types followed the ‘tissue layer’ trajectory well (dashed arrow). **f**, Model for combinatorial coding of the body’s internal space in VSNs using a 2D genetic matrix.

We also discovered a second orthogonal trajectory and identified its associated DEGs, which include *Gpr65*, *Sst*, *Trpv1*, *Drd2* and *Agtr1a* (Fig. 2a, Extended Data Fig. 3j). To determine whether this second trajectory also encodes essential interoceptive information, we labelled DEG^+^ VSNs through nodose ganglion injection^11^ of AAV-FLEX-tdTomato in corresponding Cre mouse lines (hereafter referred as DEG^tdT^ mice) and examined the innervation of tdTomato^+^ sensory fibres in whole-mount cleared organs. Notably, VSNs along this second trajectory project to different tissue layers (Extended Data Fig. 3k): *Gpr65*^+^ VSNs projected almost exclusively to the innermost mucosal layer in the oesophagus, stomach and duodenum but were largely absent from the heart and lung; *Sst*^+^ VSNs similarly innervated the mucosal layer mainly in the stomach around the pyloric sphincter; *Drd2*^+^ VSNs heavily terminated in the middle muscular layer across all gastrointestinal organs and the heart; and *Agtr1a*^+^ VSNs mostly terminated in the outer myenteric or epicardial layers composed of connective collagenous matrix. To better quantify the locations of VSN endings along the surface–lumen axis of organs, we gave each tissue layer an index (innermost mucosal or epithelial layer, 0; middle muscular layer, 1; outermost connective tissue layer, 2) (Fig. 2a) and calculated the average tissue layer index score for DEG^+^ VSNs in each examined organ (Methods). The tissue layer index scores of DEG^+^ VSNs correlated well with their relative positions along this trajectory (‘tissue layer’ trajectory score; Fig. 2a–c), demonstrating that the second trajectory we identified encodes the tissue layer information of VSN endings. Notably, within the same tissue layer, VSNs with lower trajectory scores—such as *Sst*^+^ and *Gpr65*^+^ VSNs—terminated significantly deeper than *Trpv1*^+^ VSNs with higher trajectory scores (Extended Data Fig. 3l, m), suggesting that this trajectory also encodes the relative position of VSN endings along the organ’s surface–lumen axis; this implies that vagal afferents may follow morphogen gradients into deeper tissue layers.

**Extended Data Fig. 4 |:**
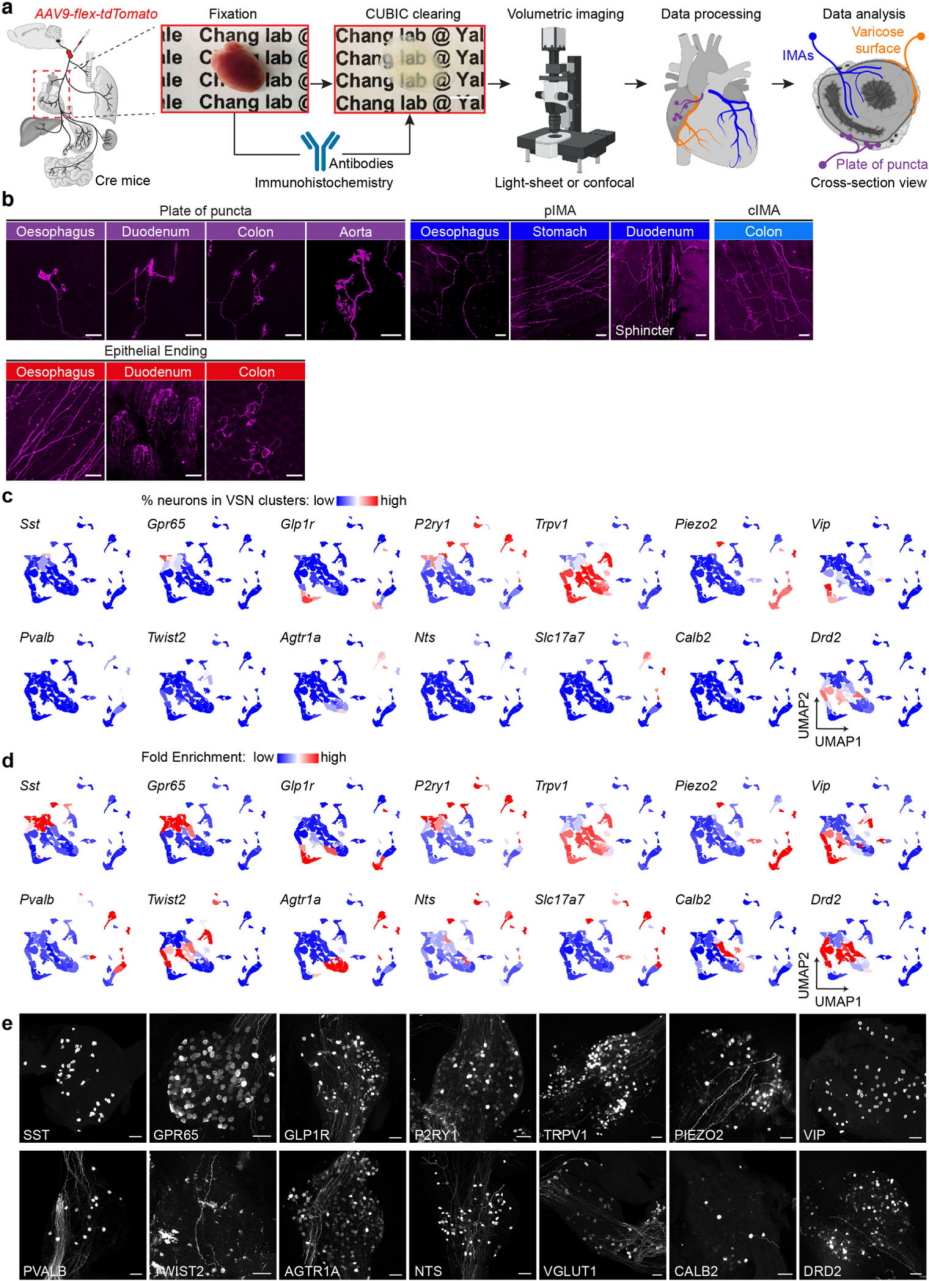
AAV-guided anatomical tracing using various Cre mouse lines. **a**, Schematic illustration of imaging DEG^+^ VSN endings in intact cleared visceral organs using nodose ganglia injection of AAV-FLEX-tdTomato in corresponding Cre mouse lines. A mouse heart before and after CUBIC clearing was shown. Leftmost panel adapted with permission from ref. ^11^; remainder of the illustration was adapted from BioRender.com. **b**, Stereotypical VSN sensory endings characterized in various visceral organs in Vglut2^tdT^ mice. **c**, UMAP plots of VSN clusters, coloured by the percentage of gene^+^ VSNs in each cluster. **d**, UMAP plots of VSN clusters, coloured by fold enrichment, calculated as the percentage of gene^+^ VSNs in the target cluster normalized by the overall percentage of gene^+^ VSNs in all clusters. **e**, Representative images of vagal ganglia from the following mice: *Sst-Cre;lox-L10-GFP*, Gpr65^tdT^, Glp1r^tdT^, P2ry1^tdT^, Trpv1^tdT^, Piezo2^tdT^, *Vip-Cre;lox-L10-GFP*, *Pvalb-Cre;lox-tdT*, Twist2^tdT^, Agtr1a^tdT^, Nts^tdT^, *Vglut1-Cre;lox-ChR2-eYFP*, Calb2^tdT^ and Drd2^tdT^. Scale bars: 0.5 cm (**a**), 100 μm (**b**, **e**).

We then asked whether VSNs form organ-specific ending structures in various tissue layers. Although VSN endings have been extensively characterized in individual organs^6–9,15–17^, it is unclear how they contribute to interoceptive coding. Systematic analyses of VSN terminals within the lung, heart, oesophagus, stomach, duodenum and colon in Vglut2^tdT^ mice revealed that there is a marked similarity between many stereotypical VSN ending types in the same tissue layer across visceral organs (Fig. 2d, Extended Data Fig. 4a, b), including (1) plates of terminal puncta lying in a collagenous and elastic environment; (2) varicose free endings in the fibrous adventitia; (3) parallel intramuscular arrays (pIMAs); (4) irregular muscular endings innervating muscle bundlesfacing different directions with circular parent neurites (cIMAs); (5) free endings beneath the luminal epithelial lining (absent in the heart and arteries, in which endoderm-derived epithelium is missing^36^); and (6) bud endings wrapped around specialized sensory epithelial cell clusters. Our data thus suggest that VSN ending structures are predominantly organ-independent but tightly associated with tissue layers.

**Extended Data Fig. 5 |:**
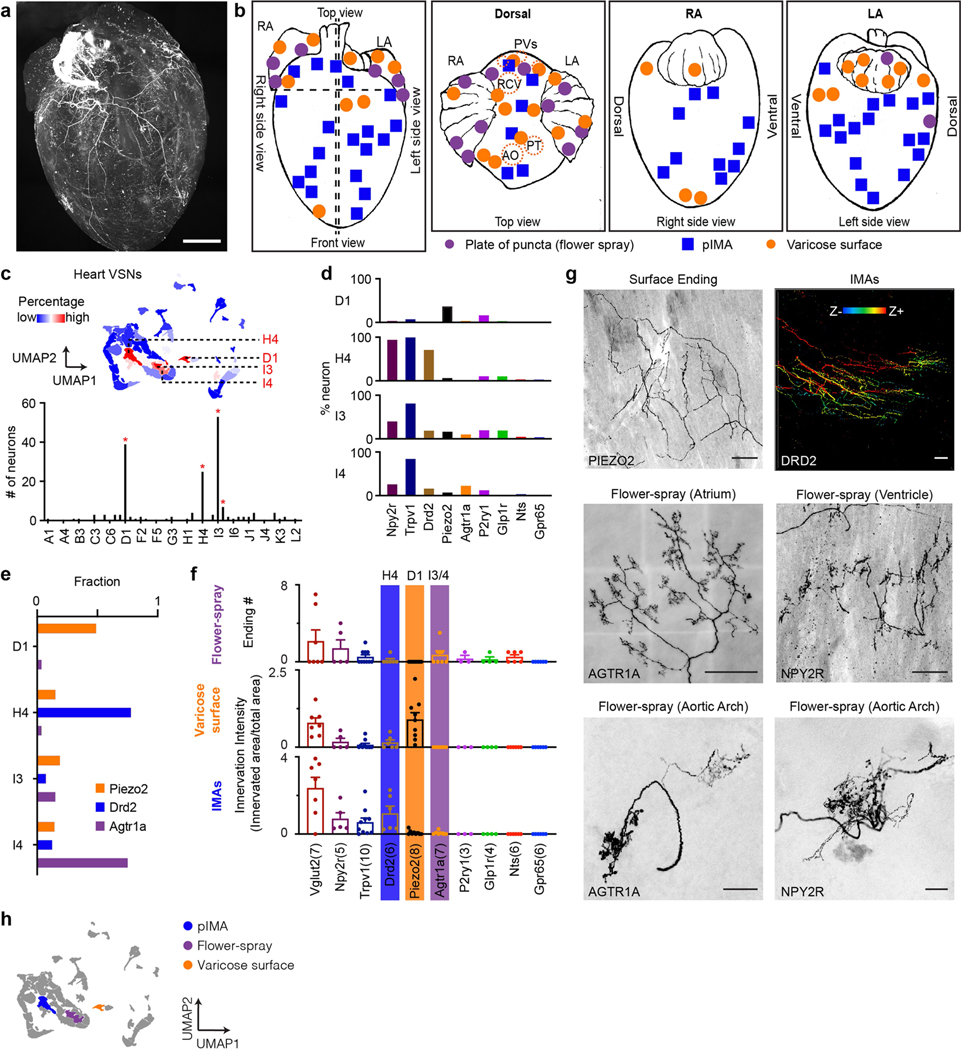
Projection-seq-guided mapping of heart VSNs. **a**, Light sheet image of a cleared heart from Vglut2^tdT^ mice showing the projection of vagal cardiac afferents. **b**, Distribution of the three vagal afferent ending types in the heart. Purple circles, plate of puncta (flower spray) endings; blue squares, parallel intramuscular arrays; orange circles, varicose surface endings. RA, right atrium. LA, left atrium. RCV, right cardiac vein. AO, aorta. PT, pulmonary trunk. **c**, 4 primary VSN clusters for heart UPB-labelled neurons, visualized on the UMAP plot (top) or column graph (bottom, red stars). **d**, Percentage of VSNs expressing indicated genes targeted in various Cre mouse lines in the 4 primary heart clusters. **e**, Fraction of DEG^+^ VSNs in indicated clusters, calculated as the number of DEG^+^ VSNs in the indicated cluster normalized by the total number of DEG^+^ VSNs in all the 4 primary heart clusters. **f**, Innervation density of heart ending types formed by indicated VSNs in corresponding Cre^tdT^ mice (mean ± SEM, n = 3–10 samples), indicating that the three VSN heart ending types are formed by different VSN subpopulations. **g**, Representative cardiac endings formed by indicated VSNs in corresponding Cre^tdT^ mice. Depth of IMAs labelled in Drd2^tdT^ mice are colour-coded. **h**, UMAP plot of VSN clusters indicating corresponding ending types in the heart. Scale bars: 1 mm (**a**), 100 μm (**g**).

**Extended Data Fig. 6 |:**
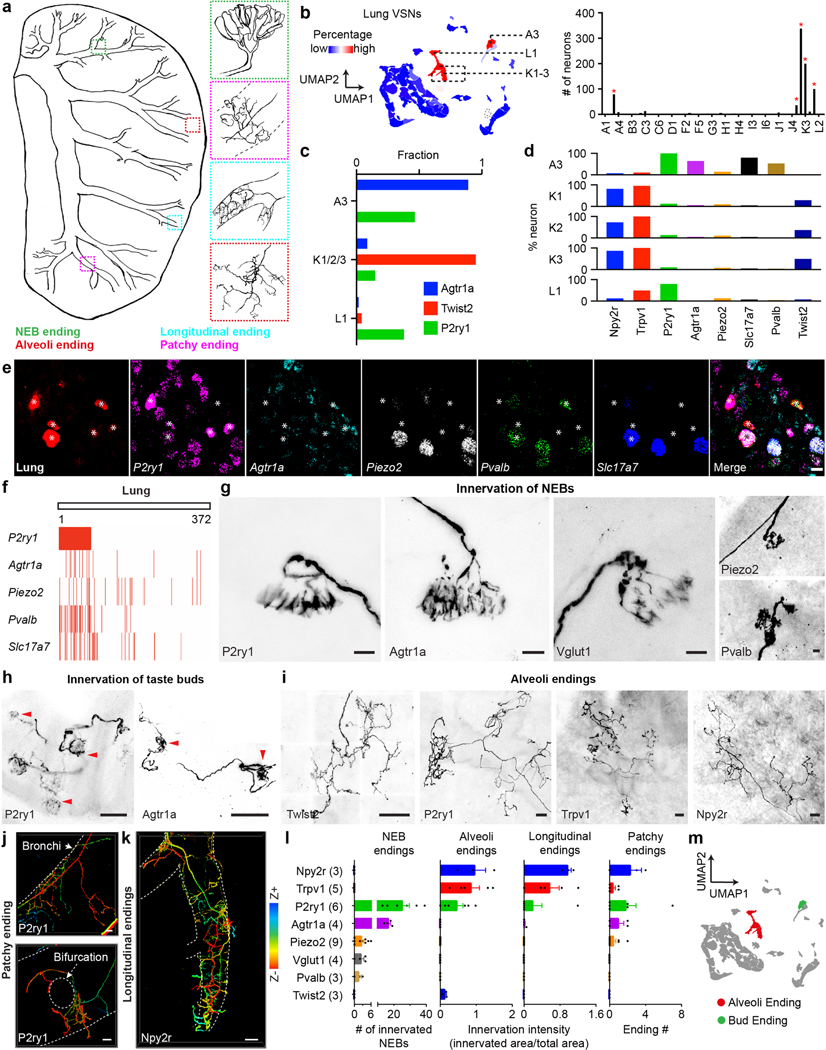
Projection-seq-guided mapping of lung VSNs. **a**, Cartoon depiction of VSN ending types in the lung. **b**, 5 primary VSN clusters for lung UPB-labelled neurons, visualized on the UMAP plot (left) or column graph (right, red stars). **c**, Fraction of DEG^+^ VSNs across indicated primary clusters. **d**, Percentage of VSNs expressing indicated genes targeted in various Cre mouse lines in indicated clusters. **e**, RNAscope HiPlex Assay for various A3 cluster marker genes as shown in (**d**) in vagal ganglia. VSNs projecting to the lung were retrogradely labelled with AAVrg and visualized using an RNAscope probe. **f**, Expression of indicated genes in retrogradely labelled lung VSNs (one neuron per column). Note that A3 cluster markers including *P2ry1*, *Agtr1a*, *Pvalb*, and *Slc17a7* were largely co-expressed in the same subset of lung VSNs. **g**, Bud endings around pulmonary neuroepithelial bodies in P2ry1^tdT^, Agtr1a^tdT^, Vglut1^tdT^, Piezo2^tdT^, and Pvalb^tdT^ mice. **h**, Bud endings (arrow heads) wrapping around taste buds in the larynx and upper oesophagus in P2ry1^tdT^ and Agtr1a^tdT^ mice (see Methods–Annotation of VSN clusters). **i**, Alveoli endings in Twist2^tdT^, P2ry1^tdT^, Trpv1^tdT^, and Npy2r^tdT^ mice. **j**, **k**, 3D projection of stacked confocal images showing patchy endings along the bronchi (top) and around bronchi bifurcations (bottom) in P2ry1^tdT^ mice (**j**) and longitudinal endings wrapping around bronchioles in Npy2r^tdT^ mice (**k**). Fibre depths are colour-coded. **l**, Innervation density of lung VSN ending types formed by indicated VSNs in corresponding Cre^tdT^ mice (mean ± SEM, number of mice as indicated). **m**, UMAP plot of VSN clusters indicating corresponding ending types in the lung. Scale bars: 20 μm (**e**, **g**), 100 μm (**h**–**k**).

**Extended Data Fig. 7 |:**
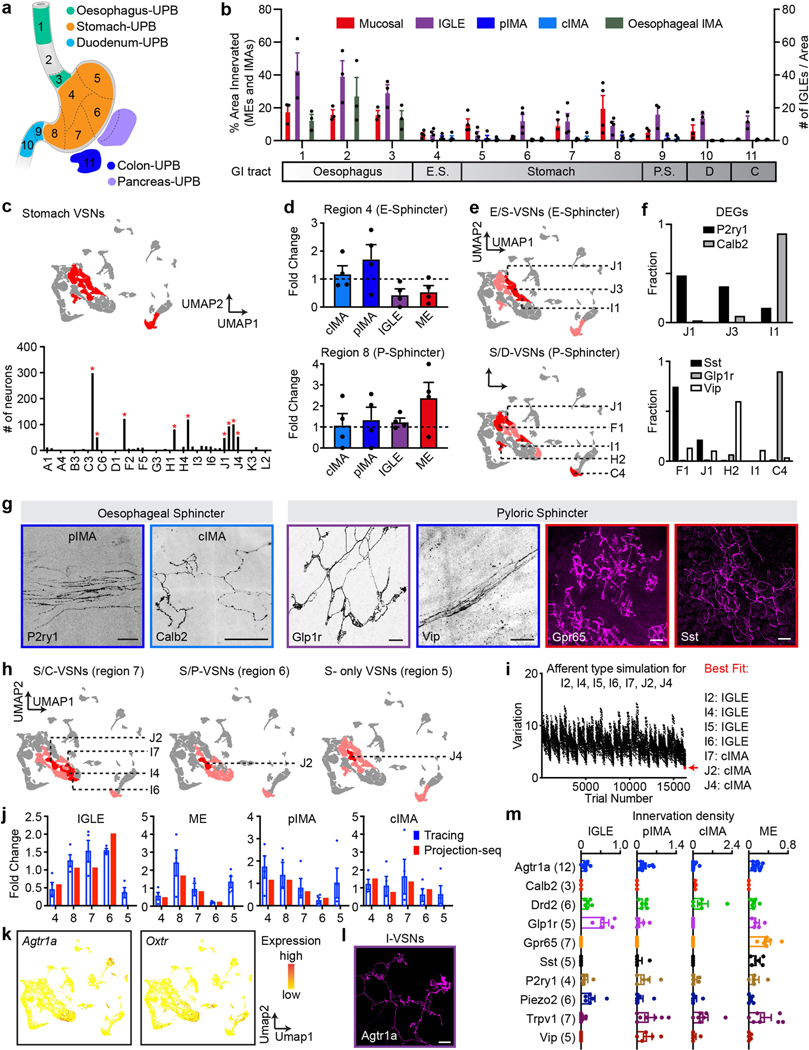
Projection-seq-guided mapping of stomach VSNs. **a**, Cartoon depiction of the 11 regions along the gastrointestinal tract with injection sites indicated. Cartoon illustration adapted with permission from ref. ^11^. **b**, Innervation intensity of various vagal afferent ending types (innervated area/total area for mucosal endings and IMAs, number of terminals/total area for IGLEs) along indicated regions on the gastrointestinal tract in Vglut2^tdT^ mice. (mean ± SEM, n = 3–4). E.S., oesophageal sphincter; P.S., pyloric sphincter; D, duodenum; C, transverse colon. Regions 1–3, upper, middle, and lower oesophagus. Regions 4–8 correlate with stomach regions as shown in (**a**). **c**, 9 primary VSN clusters for stomach UPB-labelled neurons, visualized on the UMAP plot (top, red) or column graph (bottom, red stars). **d**, Innervation density of vagal ending types around the oesophageal (top) and pyloric (bottom) sphincters, normalized to the density across the entire stomach (mean ± SEM, n = 4). **e**, UMAP plots of VSN clusters enriched for the oesophageal (top, VSNs dual labelled with oesophagus and stomach UPBs) and pyloric (bottom, VSNs dual labelled with stomach and duodenum UPBs) sphincters over other stomach regions. Light red indicates clusters containing more than 4% of dual UPB labelled VSNs. Red indicates clusters enriched (>1.05 fold) in dual UPB labelled VSNs over stomach UPB labelled VSNs. **f**, Fraction of DEG^+^ VSNs among indicated clusters, calculated as the number of DEG^+^ VSNs in the indicated cluster normalized by the total number of DEG^+^ VSNs in all shown clusters. **g**, Representative stomach endings formed by DEG^+^ VSNs around the oesophageal or pyloric sphincter in corresponding Cre^tdT^ mice. **h**, UMAP plots of VSN clusters (red) enriched for the stomach regions 5, 6, and 7 as shown in (**a**). Light red indicates clusters containing more than 4% of stomach/colon (region 7) or stomach/pancreas (region 6) dual UPB labelled, or stomach UPB single labelled (region 5) VSNs. Red indicates clusters enriched (>1.05 fold) in dual UPB labelled VSNs over stomach UPB labelled VSNs (for region 6 and 7), or in stomach UPB labelled VSNs over all other 4 groups of dual UPB labelled VSNs (for region 5). **i**, Simulation results for VSN clusters I2, I4–7, J2, and J4 (see Methods). Arrow indicates the trial with the lowest variation (red dots, ending types listed on the right). **j**, Innervation density of four VSN ending types in different stomach regions normalized to the intensity across the entire stomach. Quantified in Vglut2^tdT^ mice (blue, mean ± SEM, n = 4) or predicted by Projection-seq (red). **k**, UMAP plots of VSNs showing *Agtr1a* and *Oxtr* expression in I-VSNs. **l**, Representative stomach IGLE endings formed by I-VSNs in Agtr1a^tdT^ mice. **m**, Innervation intensity (innervated area/total area for ME, pIMA, and cIMA; number/total area for IGLE, normalized to Vglut2^tdT^) of stomach afferent ending types formed by indicated VSNs in corresponding Cre^tdT^ mice (mean ± SEM, n = 3–12). Scale bars: 100 μm.

**Extended Data Fig. 8 |:**
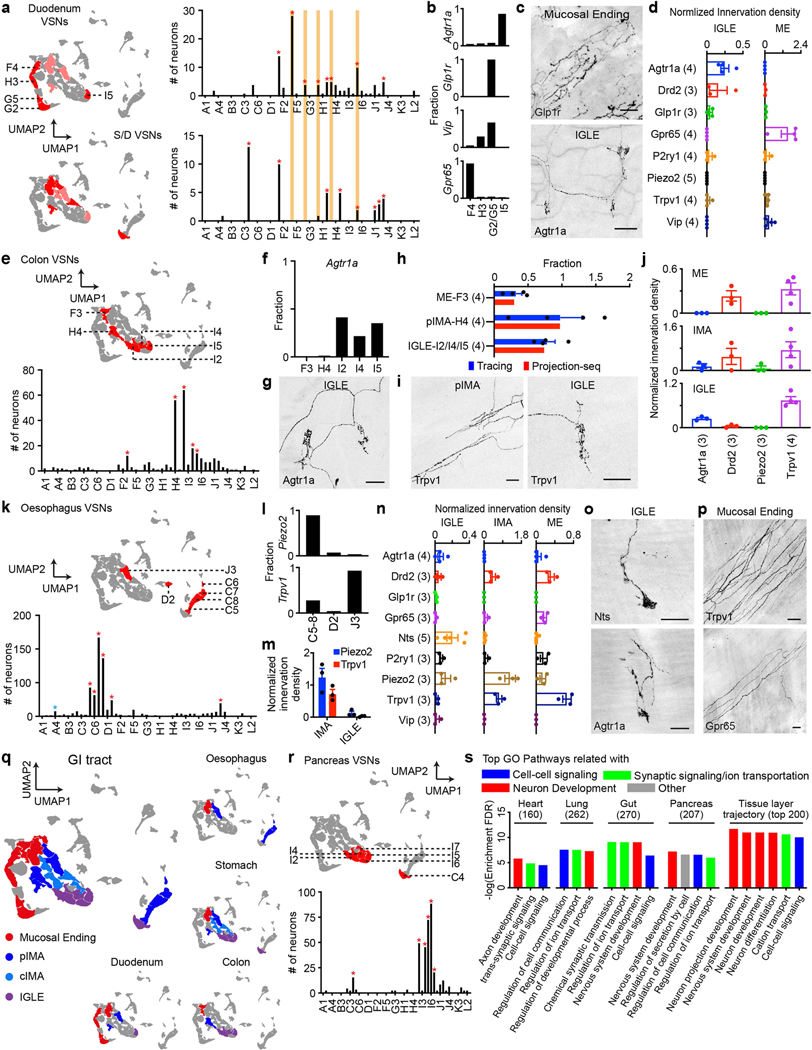
Projection-seq-guided mapping of oesophagus, duodenum and colon VSNs. **a**, 8 primary duodenum UPB-labelled VSN clusters (top) and 8 primary S/D (stomach/duodenum) dual UPB-labelled VSN clusters (bottom), visualized on the UMAP plot (left, light red indicates clusters containing more than 4% labelled VSNs; red indicates enriched clusters) or column graph (right, red stars). Five VSN clusters enriched in duodenum VSNs over S/D VSNs were labelled (top left, red) and marked with orange bars (top right). **b**, Fraction of DEG^+^ VSNs among the five clusters identified in (**a**). **c**, Representative sensory endings formed by DEG^+^ VSNs in the duodenum in corresponding Cre^tdT^ mice. **d**, Innervation intensity (innervated area/total area for ME; number/total area for IGLE, normalized to Vglut2^tdT^, same for **j**, **m**, and **n**) of duodenal afferent ending types formed by indicated VSNs in corresponding Cre^tdT^ mice (mean ± SEM, n = 3–5). **e**, 5 primary VSN clusters for colon UPB-labelled neurons, visualized on the UMAP plot (top, red) or column graph (bottom, red stars). **f**, Fraction of Agtr1a^+^ VSNs among the five clusters identified in (**e**). **g**, Colon IGLE formed in Agtr1a^tdT^ mice. **h**, Innervation density of Trpv1^+^ VSN ending types in the colon, determined via anatomical tracing in Trpv1^tdT^ mice (blue, innervated area/total area for ME and pIMA; number/total area for IGLE, normalized to Vglut2^tdT^, mean ± SEM, n = 4) or predicted by Projection-seq (red). **i**, Representative colon IMA and IGLE endings formed in Trpv1^tdT^ mice. **j**, Innervation intensity of colon afferent ending types formed by indicated VSNs in corresponding Cre^tdT^ mice (mean ± SEM, n = 3–4). **k**, 6 primary VSN clusters for oesophagus UPB-labelled neurons, visualized on the UMAP plot (top, red) or column graph (bottom, red stars). A4-VSNs (1.68%, blue star) was characterized to form rare bud endings wrapping around taste buds in the upper oesophagus (see Extended Data Fig. 6). **l**, Fraction of *Piezo2*^+^ and *Trpv1*^+^ VSNs among the five clusters identified in (**k**). **m**, Innervation intensity of oesophageal afferent ending types formed in Piezo2^tdT^ and Trpv1^tdT^ mice (mean ± SEM, n = 3). **n**, Innervation intensity of oesophageal afferent ending types formed by indicated VSNs in corresponding Cre^tdT^ mice (mean ± SEM, n = 3–5). **o**, Representative oesophageal IGLEs formed in Nts^tdT^ (top) and Agtr1a^tdT^ (bottom) mice. **p**, Representative oesophageal mucosal endings formed in Trpv1^tdT^ (top) and Gpr65^tdT^ (bottom) mice. **q**, UMAP plots of VSN clusters indicating corresponding ending types in the oesophagus, stomach, duodenum, and colon, and all examined gastrointestinal organs. **r**, 6 primary pancreas UPB-labelled VSN clusters, visualized on the UMAP plot (left, red) or column graph (right, red stars). **s**, Top GO pathways of DEGs in heart, lung, gut, and pancreas VSNs, and along the tissue layer trajectory, coloured by physiological functions. Number of DEGs used are indicated. FDR, false discovery rate. Scale bars: 100 μm.

To further ask whether morphologically stereotypical VSN endings across organs have similar molecular identities, we performed Projection-seq-guided anterograde tracing to determine the genetic signatures of VSN ending structures. For each organ or anatomical region (oesophageal and pyloric sphincters), we (1) identified enriched VSN clusters and their DEGs on the basis of Projection-seq data; (2) labelled DEG^+^ VSNs by nodose infection of AAV-FLEX-tdTomato in corresponding Cre mice; and (3) determined the sensory structures formed by DEG^+^ VSNs in the target organ or region using whole-organ clearing and volumetric imaging (Fig. 2e, Extended Data Fig. 4). With this approach, we determined the molecular identity of VSN endings in the heart, lung, stomach, oesophagus, duodenum and colon (Extended Data Figs. 5–8; see annotation details in Methods). VSN clusters forming various VSN ending types followed the tissue layer trajectory well (Fig. 2e), showing that from both an anatomical and a genetic perspective, VSN ending structures are specialized for their local tissue layer environments rather than for visceral organs.

The fact that ‘visceral organ’ and ‘tissue layer’ trajectories are orthogonal shows that they are coded in parallel rather than hierarchically. DEGs among VSNs that innervate different visceral organs and along the ‘tissue layer’ trajectory were both predominantly linked with Gene Ontology (GO) biological processes for neuron development, cell–cell signalling and synaptic signalling or ion transportation (Extended Data Fig. 8s), suggesting that VSN specifications for visceral organs and tissue layers are achieved using genes that have similar biological functions. Compared to hierarchical coding, a parallel coding structure can effectively reduce the number of genes required, which aligns with the efficient coding hypothesis that sensory neurons use as few resources as possible to code the maximum information^37,38^. Together, our results reveal a two-dimensional genetic representation of the body’s internal space in VSNs that codes the precise anatomical location of interoceptive signals (Fig. 2f).

## A third ‘stimulus modality’ coding dimension

Next, we sought to understand how stimulus modality is coded in VSNs. VSNs might develop visceral-organ- or tissue-layer-specific sensory mechanisms or use more generalized mechanisms for similar stimulus modalities across the body. However, as VSN identity cannot be accurately determined using only one or two marker genes^17,18^, it is challenging to uncover stimulus modality coding without more systems-based approaches.

**Extended Data Fig. 9 |:**
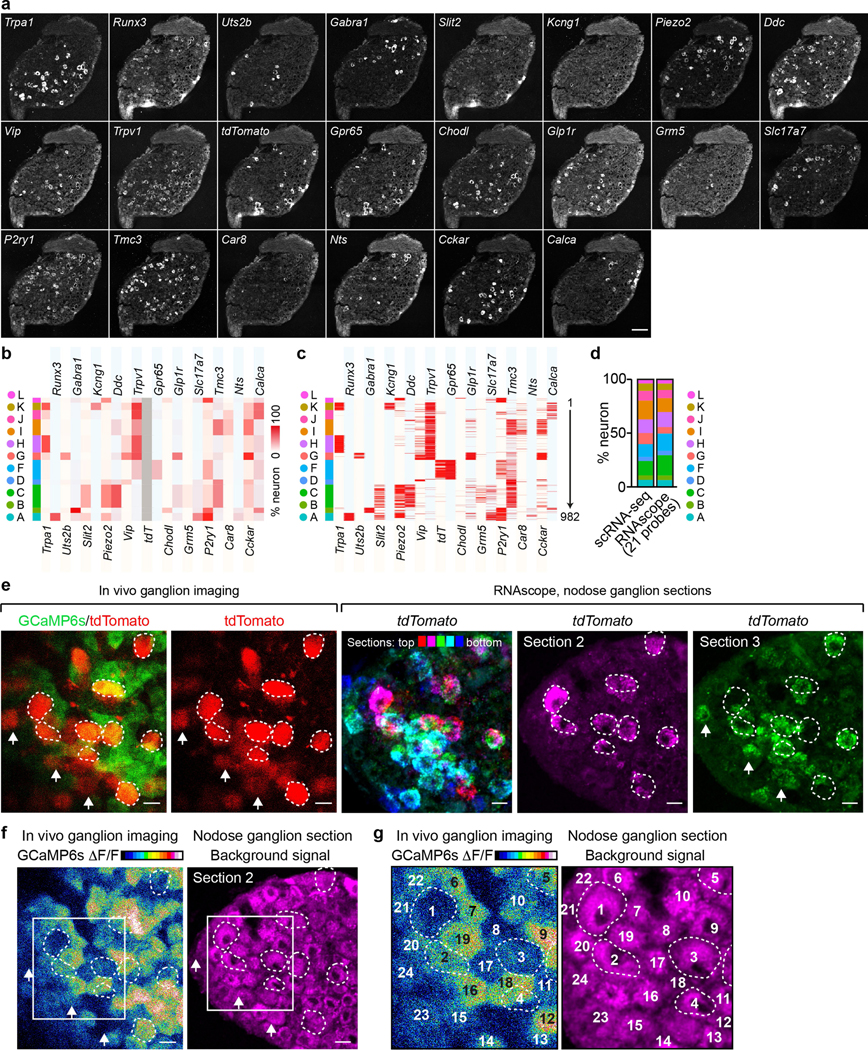
Development of vCatFISH analysis. **a**, RNAscope HiPlex Assay of VSNs for the indicated 22 genes in cryo-sectioned vagal ganglia after in vivo calcium imaging. Scale bar: 100 μm. **b**, Percentage of neurons expressing the indicated 22 genes in 11 VSN subpopulations (A-L, colour-coded, from bottom to top) in Projection-seq data (n = 12,583). **c**, VSN subpopulations (A-L, colour-coded, from bottom to top) determined using expression of indicated genes from RNAscope HiPlex Assays. One neuron per row as indicated on the right (n = 982, 136 (13.8%) of which were tdTomato^+^). **d**, Cumulative percentage of neurons revealed by Projection-seq or RNAscope in 11 VSN subpopulations. **e**, GCaMP6s and tdTomato signals recorded from in vivo ganglion imaging (left) and RNAscope signal against *tdTomato* in a series of cryosections (depth colour-coded) of the same nodose ganglion (right), showing a perfect registration of tdTomato^+^ VSNs between in vivo ganglion imaging and post-hoc RNAscope cryosections. Dashed lines indicate bright tdTomato^+^ cells in the imaging plane. Arrows indicate weak tdTomato^+^ cells off (below) the imaging plane. **f**, GCaMP6s signals (ΔF/F, colour-coded) as in (**e**, left) showing VSNs from in vivo ganglion imaging (left) and increased background signal from RNAscope against *Grm5* showing all VSNs from post-hoc nodose ganglion cryosections (right). Dashed circles and arrows indicate bright and weak tdTomato^+^ cells as shown in (**e**). **g**, Zoom-in images from the box in (**f**), with numbered VSNs showing registration between in vivo ganglion imaging and post-hoc RNAscope analysis. Scale bars: 100 μm (**a**), 20 μm (**e**, **f**).

**Extended Data Fig. 10 |:**
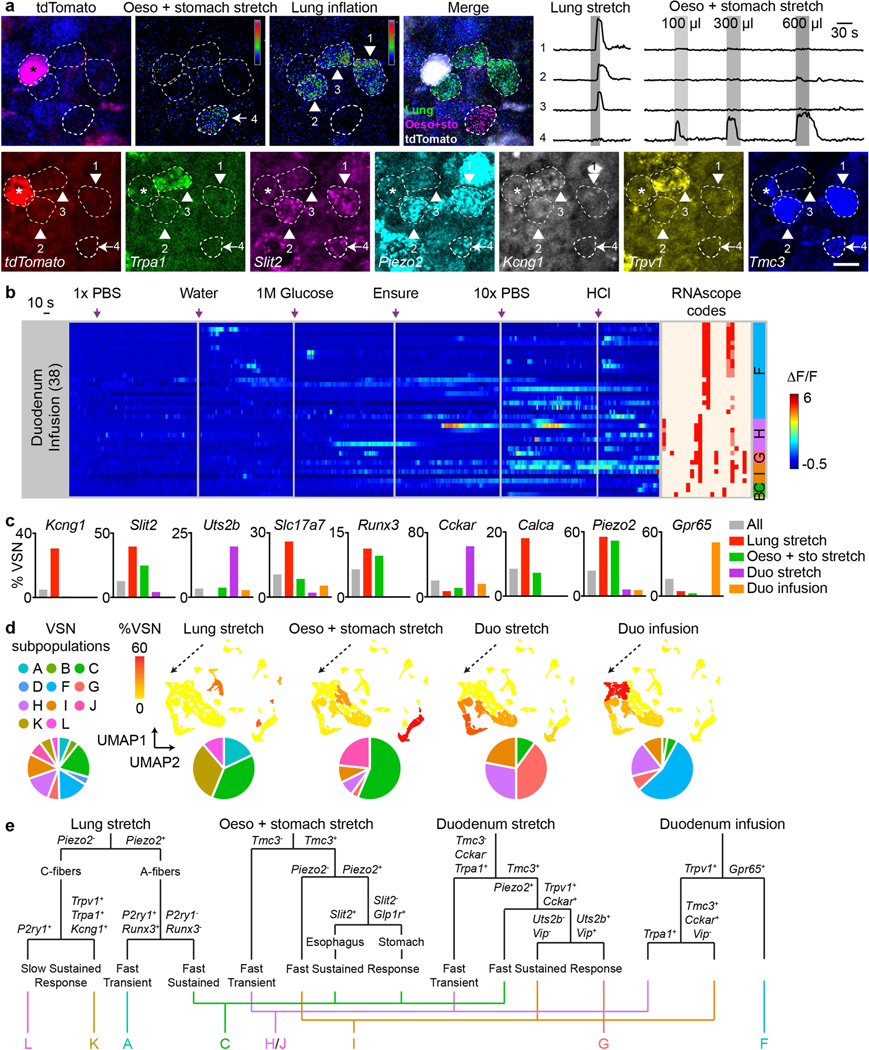
vCatFISH reveals genetic identity of VSNs responsive to various body stimuli. **a**, Representative images showing VSN GCaMP responses and expression of marker genes. (top) A Gpr65^+^ VSN (tdTomato labelled, magenta; background GCaMP6s signal in blue showing the shape of all VSNs) and GCaMP6s responses (ΔF/F, colour-coded) to indicated stimuli in a representative vagal ganglion of Gpr65^tdT^-GCaMP6s mice. Responses (ΔF/F) of indicated VSNs are shown on the right. (bottom) RNAscope analysis of the same VSNs for indicated genes in vagal ganglion cryosections. Scale bars: 20 μm. **b**, Time-resolved responses (ΔF/F, colour-coded) of 38 single VSNs to indicated chemicals (saline, water, 1 M glucose, Ensure, 10 x Phosphate-Buffered Saline (PBS), 150 mM HCl, 100 μl) injected into duodenal lumen in Gpr65^tdT^-GCaMP6s mice (n = 5). RNAscope codes are in the same sequence as shown in Fig. 3b and Extended Data Fig. 9b, c. Annotated subpopulations (colour-coded) for responsive VSNs are indicated on the right. **c**, Genetic compositions of all VSNs (grey) or VSN cohorts activated by various indicated stimuli (colour-coded), showing by the percentage of neurons expressing indicated genes. **d**, Subpopulations of VSNs activated by indicated stimuli. (top), UMAP plots of VSN subpopulations (A-L), coloured by the percentage of neurons activated by the indicated stimulus in each subpopulation, showing that VSNs responsive to stimuli from thoracic and abdominal organs follow the ‘visceral organ’ trajectory (dashed arrow). (bottom), Pie chart indicating the percentage of VSN subpopulations in all VSNs or VSN cohorts responding to indicated stimuli. **e**, Summary of VSN groups responsive to diverse stimuli, with key marker genes, response patterns, and VSN subpopulations determined using RNAscope, showing modular VSN sensory units across multiple visceral organs.

**Fig. 3 |:**
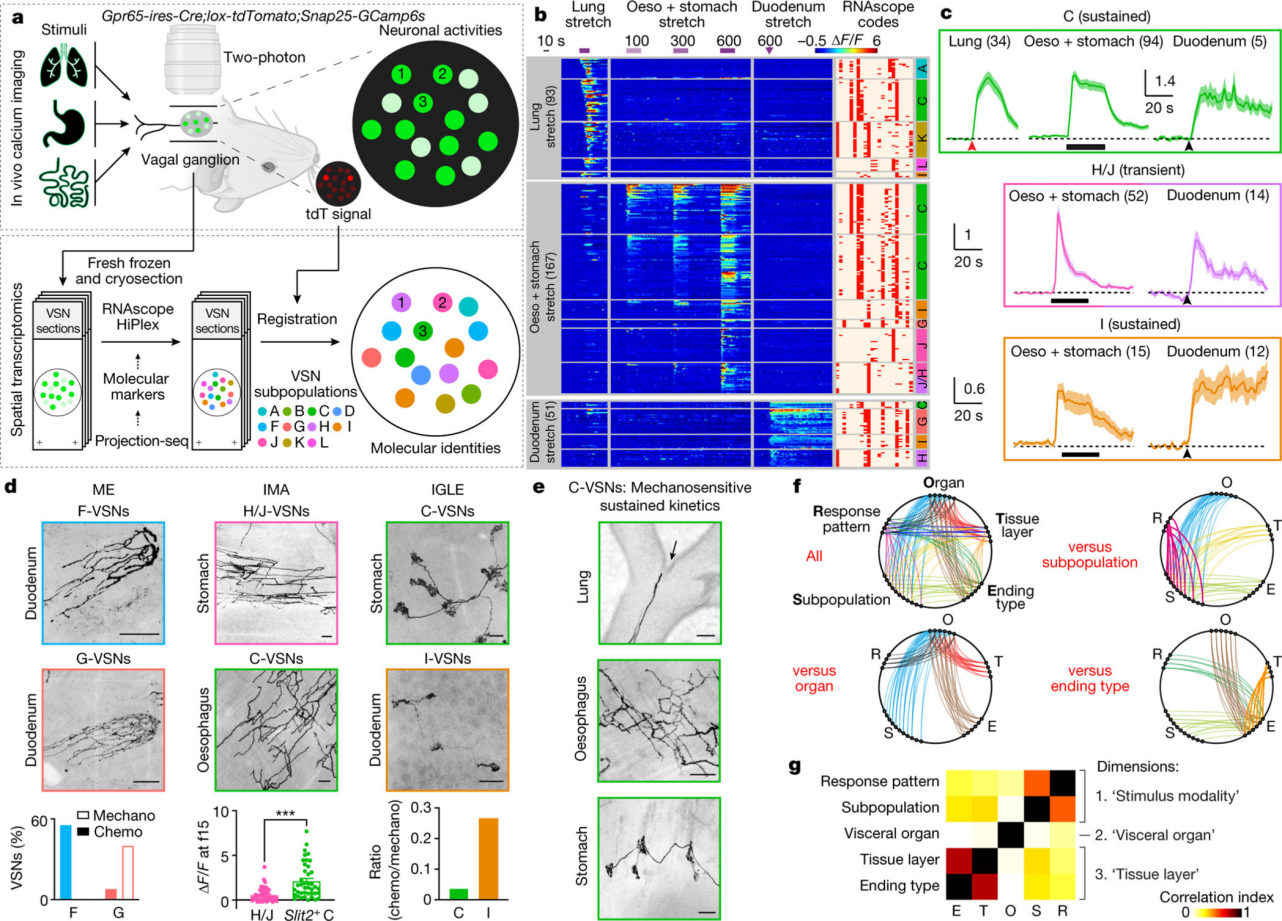
vCatFISH analysis reveals a third ‘stimulus modality’ coding dimension in VSNs. **a**, Schematic illustration of vCatFISH analysis. Organ illustrations were adapted from BioRender.com. **b**, Time-resolved responses (Δ*F*/*F*; colour-coded) of 311 single VSNs to the indicated stimuli (lung stretch: oxygen, 600 ml min^−1^, 20 s; oesophagus and stomach stretch: saline, 100, 300, 600 μl, 30 s; duodenum stretch: saline, 600 μl) in Gpr65^tdT^-GCaMP6s mice (*n* = 6) with RNAscope codes (Extended Data Fig. 9c) and annotated subpopulations (A–L). **c**, VSN subpopulations show stereotypical response patterns across organs (Δ*F*/*F*; mean ± s.e.m.; number of VSNs as indicated). Lung stretch responses were aligned at the activation frame (red arrowhead) to reveal response kinetics. Bars represent oesophagus and stomach stretch; black arrowheads represent duodenal stretch (saline, 600 μl). **d**, VSN endings are uncoupled from response patterns. Left, F-VSNs (Gpr65^tdT^) and G-VSNs (Vip^tdT^) form indistinguishable mucosal villi endings (ME) in the intestine but respond to different sensory cues (bottom, 5 mice). Middle, pIMAs formed by H/J-VSNs (P2ry1^tdT^) and C-VSNs (Piezo2^tdT^) have distinct response kinetics to oesophagus and stomach stretch (bottom, Δ*F*/*F* at post-activation frame 15 (f15), mean ± s.e.m., 5 mice, ***P < 0.001, *P* = 7.4 × 10^−7^, two-tailed *t*-test). Right, IGLEs formed by C-VSNs (Piezo2^tdT^) and I-VSNs (Agtr1a^tdT^) have different preferences between mechanical and chemical stimuli (bottom, 5 mice). **e**, Different ending types formed by C-VSNs in the lung, oesophagus and stomach (Piezo2^tdT^). Scale bars, 100 μm (**d**, **e**). **f**, Correlation maps showing all mapped connections among various VSN characteristics. One-to-one connection pattern indicates perfect correlation; all-to-all connection pattern indicates no correlation. Magenta (top right) and gold (bottom right) connections indicate strong correlations between VSN subpopulation and response pattern and between tissue layer and ending type. **g**, Correlation index between pairs of VSN characteristics, calculated on the basis of the number and pattern of connections shown in **f**, showing three independent feature-coding dimensions in VSNs.

We developed a technique named ‘vagal calcium imaging transformed fluorescence in situ hybridization’ (vCatFISH) (Fig. 3a). First, the neuronal activity in hundreds of VSNs was simultaneously imaged in vivo in response to various body stimuli including lung inflation, oesophagus and stomach stretch, intestine stretch and a series of chemical challenges delivered to the intestinal lumen (water, salt, nutrient and acid) using *Gpr65-ires-Cre;lox-tdTomato;Snap252a-GCaMP6s-D* (Gpr65^tdT^-GCaMP6s) mice. VSN identity was then determined post hoc in cryo-sectioned nodose ganglia using RNAscope against 21 marker genes that together faithfully represent VSN subpopulations (Extended Data Fig. 9a–d). Finally, tdTomato^+^ cells were used as landmarks to register VSNs between calcium imaging and RNAscope assays (Extended Data Figs. 9e, 10a). The proper density (13.8%) and scattered distribution of tdTomato^+^ VSNs in Gpr65^tdT^-GCaMP6s mice ensure the successful registration of tdTomato^−^ VSNs. In total, 57.5% (349/607) responsive VSNs were unambiguously registered from six mice (Fig. 3b, Extended Data Fig. 10b, c). VSNs responding to stimuli applied to organs along the body’s rostral–caudal axis closely followed the ‘visceral organ’ trajectory (Extended Data Fig. 10d). The vCatFISH results are consistent with previous reports regarding individual marker genes^11–13,39,40^, and provide the precise identity of responsive VSNs.

**Extended Data Fig. 11 |:**
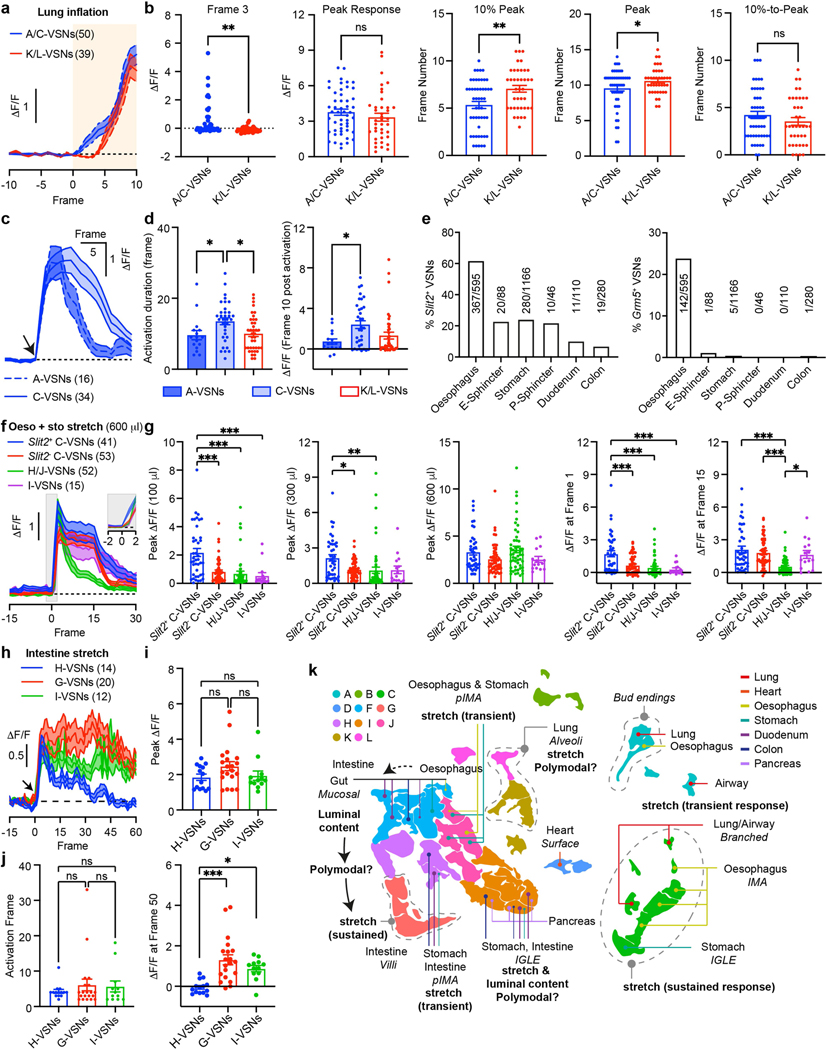
VSN subpopulations specify response patterns. **a**, **b**, GCaMP responses to lung stretch (**a**) and quantification of activation kinetics (**b**) in A/C-VSNs (n = 50) and K/L-VSNs (n = 39) from 5 mice. **c**, GCaMP responses with activation frame (arrow) aligned showing C-VSNs (n = 34) and A-VSNs (n = 16) have different adaptation rates to lung stretch. **d**, Quantification of adaptation kinetics as shown in (**c**). **e**, Percentage of UPB-labelled VSNs from indicated gastrointestinal organs/regions expressing *Slit2* (left) and *Grm5* (right) in Projection-seq data. **f**, **g**, GCaMP responses to oesophagus/stomach stretch (600 μl) (**f**) and quantification (**g**) in *Slit2*^+^ C- (n = 41), *Slit2*^−^ C- (n = 53), H/J- (n = 52), and I- (n = 15) VSN subpopulations from 6 mice. *Slit2*^+^ C-VSNs were activated significantly faster with lower activation threshold, consistent with them being mechano-sensors in the oesophagus. **h**–**j**, GCaMP responses (**h**) to intestine stretch (saline, 600 μl, arrow) and quantification of response magnitudes (**i**) and activation kinetics (**j**) in H- (n = 14), G- (n = 20), and I- (n = 12) VSN subpopulations from 5 mice. **k**, Comprehensive UMAP plot of VSNs from integration of Projection-seq, vCatFISH, and Projection-seq-guided anterograde tracing analyses. VSN subpopulations (A-L) are colour-coded as in Fig. 1b. Organs are indicated by coloured lines. Ending structures are indicated using italic. Sensory properties are labelled in bold. Dashed arrow indicates a continuous representation of gut organs along the gastrointestinal tract. Arrow indicates a potential gradual shift of sensory inputs from chemical to mechanical sensation in the intestine. Some VSN clusters with similar characteristics are indicated by dashed lines. Imaging frequency: 1.72 second/frame. mean ± SEM. *p < 0.05, **p < 0.01, ***p < 0.001, two-tailed *t*-test for (**b**), one-way ANOVA and Tukey’s multiple comparisons test for (**d**, **g**, **i**, **j**). P values for shown comparisons from left to right are as follows: **b**, 0.002, 0.3057, 0.0017, 0.0492, 0.2255; **d**, 0.043, 0.015, 0.021; **g**, 4.1 × 10^−6^, 4.6 × 10^−7^, 1.7 × 10^−4^, 0.01084, 0.00570, 1.3 × 10^−4^, 2.3 × 10^−6^, 0.0004, 1.3 × 10^−6^, 3.0 × 10^−5^, 0.0363; **i**, 0.1623, 0.9692, 0.2918; **j**, 0.6378, 0.8140, 0.9766, 5.2 × 10^−5^, 0.0159.

Although VSNs in different subpopulations typically show distinct response patterns, all of the examined stimuli activated multiple subpopulations of VSNs (Fig. 3b, Extended Data Fig. 10b, e), indicating that VSN subpopulations are primed for an individual decomposed stimulus instead of coding complex physiological changes directly. Several lung-stretch-sensitive VSN types were identified. *Piezo2*^+^ VSNs (55.1%) were activated significantly faster than *Piezo2*^−^ K/L-VSNs (Extended Data Fig. 11a, b). *Piezo2*^+^*P2ry1*^−^ C-VSNs exhibited a rapid and sustained response, whereas *Piezo2*^+^*P2ry1*^+^ A-VSNs responded more transiently (Extended Data Fig. 11c, d), providing evidence that different *Piezo2*^+^ subtypes have distinct response kinetics. For oesophagus- or stomach-stretch-sensitive VSNs, *Slit2* or *Grm5* and *Glp1r* differentially defined oesophagus and stomach C-VSNs (Extended Data Fig. 11e–g). C-VSNs (56.3%) and I-VSNs (9.0%) had sustained stretch responses, whereas H/J-VSNs (34.1%) responded only transiently (Extended Data Fig. 11f, g). Of note, VSNs sensitive to intestinal stretch were predominantly *Piezo2*^−^ (94.1%). *Uts2b*^+^*Vip*^+^*Glp1r*^+^*Cckar*^+^ G-VSNs represented a large fraction (39.2%) of intestinal stretch responders (Extended Data Fig. 11h–j), with little response to various intestinal luminal stimuli (7.9%) or mechanical challenges in other organs (lung, 0%; intestine, 3.6%), whereas I-VSNs responded to both chemical challenges to intestinal lumen and mechanical stimuli in several organs. Our results thus show that—unlike what the multi-sensor theory proposes^41^, that fibres with distinct response patterns originate from the same VSNs— response heterogeneity is generated by different subpopulations of VSNs.

VSNs in the same subpopulation often responded to a similar stimulus across multiple visceral organs (Fig. 3c). *Piezo2*^+^ C-VSNs responded to stretch in the lung, oesophagus, stomach and duodenum with similar sustained kinetics. By contrast, H-/J-VSNs showed transient response kinetics to stretch in organs along the gastrointestinal tract (Fig. 3c, Extended Data Fig. 11), suggesting that they are well suited to the detection of gastrointestinal dynamics. I-VSNs responded broadly to both stretch in many gastrointestinal organs and various intestinal luminal stimuli, indicating that they are polymodal sensors. Thus, our data show that instead of developing specialized sensory mechanisms for individual body–brain pathways, VSNs are organized into modular sensory units to code categorized stimulus modalities across visceral organs.

**Extended Data Fig. 12 |:**
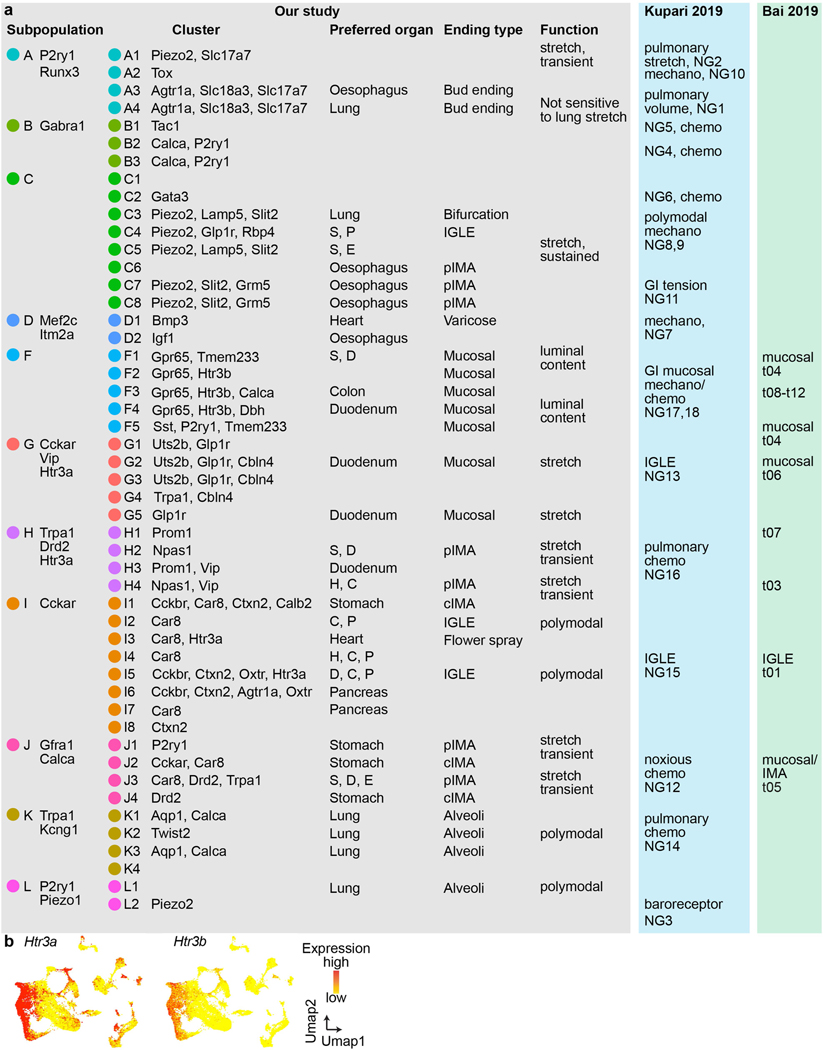
A comprehensive road map of genetically defined VSN subpopulations. **a**, A comprehensive comparison of various characteristics of VSN clusters revealed between our data and previously published results. DEGs for VSN subpopulations (left column) and clusters (second to left column) are listed. **b**, UMAP plot of VSNs, coloured by expression of *Htr3a* (left) and *Htr3b* (right) in VSNs.

We then integrated vCatFISH results with Projection-seq-guided anterograde tracing data and generated a comprehensive road map (Extended Data Figs. 11k, 12). Unlike DRG neurons, which sense diverse somatosensory cues through highly specialized afferent terminals^42^, VSN response patterns are not well correlated with their ending structures. VSNs with the same ending structures often show multiple response patterns (Fig. 3d), suggesting that they could be primed for different stimulus modalities. For example, morphologically indistinguishable intestinal villi endings can sense luminal contents (F-VSNs) or mechanical changes (G-VSNs). Notably, mechanosensitive *Piezo2*^+^ enterochromaffin cells in the intestinal villi convert force into serotonin release^43^, and the serotonin receptors *Htr3a* and *Htr3b* are highly expressed in G-VSNs^16,18^ (Extended Data Fig. 12), raising the question of whether G-VSNs are intrinsically mechanosensitive. Our data also provide functional evidence that IMAs are mechanosensitive. Genetically distinct IMAs have different response kinetics: transient (H/J-VSNs) or sustained (C-VSNs). Intraganglionic laminar endings (IGLEs) could be mechanosensitive (C-VSNs) or polymodal (I-VSNs); the latter would be ideal sensors for food ingestion, consistent with their roles in food intake^16^. Although IGLE mechanosensitivity is thought to be intrinsic^44^, luminal sensation in IGLEs must be indirect, probably through communication with enteric neurons. It is also worth noting that many more VSN types could be polymodal as the appropriate stimulus may not have been examined. On the other hand, the same VSN sensory unit, such as *Piezo2*^+^ C-VSNs, could form different ending structures in different organs (oesophagus, stomach and lung) (Fig. 3e), suggesting that irrespective of ending structures, VSNs use the same sensory mechanism to monitor similar interoceptive signals from different locations. Thus, our data show that VSN endings are not specialized for coding stimulus modality. Notably, *Piezo2*^+^ C-VSNs that recapitulate the classical slowly adapting receptors terminate at bronchi bifurcations, where the highest shear stress occurs during inspiration^45^. Together, our results identify a third ‘stimulus modality’ coding dimension in VSNs.

## A multidimensional coding scheme in VSNs

To better quantify the relationship between VSN characteristics, we systematically mapped all characterized connections among the five VSN characteristics (Fig. 3f). In this model, a perfect correlation (index = 1) between two characteristics would have a ‘one-to-one’ pattern with the minimum number of possible connections, whereas a complete uncorrelation (index = 0) would have an ‘all-to-all’ pattern with the maximum number of possible connections. We thus calculated the correlation index between each pair on the basis of the number and pattern of connections (Fig. 3g). This quantitative analysis further demonstrates key organization principles of the vagal interoceptive system: (1) VSN endings are tightly linked with tissue layers but not specialized for visceral organs and not well correlated with response patterns, indicating that VSN ending structures are primarily developed for adaptation to local tissue layers across organs; (2) VSN subpopulations defined by unbiased single-cell clustering show much stronger correlation with response patterns than with visceral organs or tissue layers, suggesting that they are more primed to code stimulus modalities; and (3) visceral organ, tissue layer and sensory modality—the three most important features that together define the physiological role of an interoceptive signal—are coded in independent dimensions in VSNs. With this coding strategy, VSNs are specified into only a few groups for each dimension, but in the combination of three dimensions, a large number of parallel pathways are generated to ensure precise and effective body-to-brain signal communication. Together, our results reveal a fundamental multidimensional coding architecture of the vagal interoceptive system.

## Complex VSN projections in the brainstem

**Extended Data Fig. 13 |:**
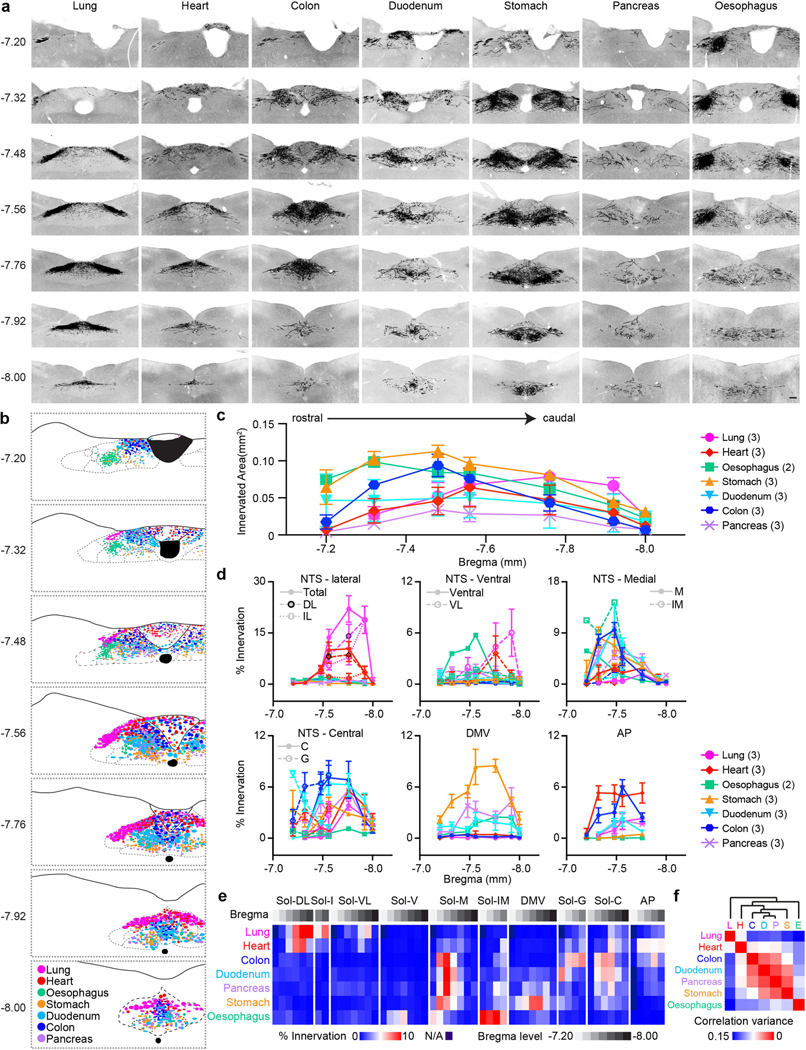
Central targets of VSNs innervating various visceral organs. **a**, Representative brainstem images at indicated Bregma levels containing central projections of VSNs retrogradely labelled from indicated visceral organs using *AAVrg-tdTomato*. Scale bar: 100 μm. **b**, Distribution of central VSNs terminals retrogradely labelled from indicated visceral organs (colour-coded) along the rostral-caudal axis. **c**, Brainstem area innervated by VSNs from indicated organs along the rostral-caudal axis (mean ± SEM, number of mice: 2 (oesophagus), 3 (others)). **d**, Percentage innervation, calculated as vagal afferent fluorescence in indicated subnuclei normalized to the fluorescence in all vagal afferent targets, for various brainstem subnuclei along the rostral-caudal axis (mean ± SEM, n as in **c**). **e**, Quantitative analysis of innervation density in indicated brainstem regions from Bregma −7.2 mm to −8.0 mm by VSNs from various visceral organs, expressed as fluorescence in indicated area/fluorescence in total area (mean, colour-coded, n as in **c**). **f**, Correlation variance matrix among organ pairs showing that VSNs from functionally related organs terminated in similar brainstem regions.

**Fig. 4 |:**
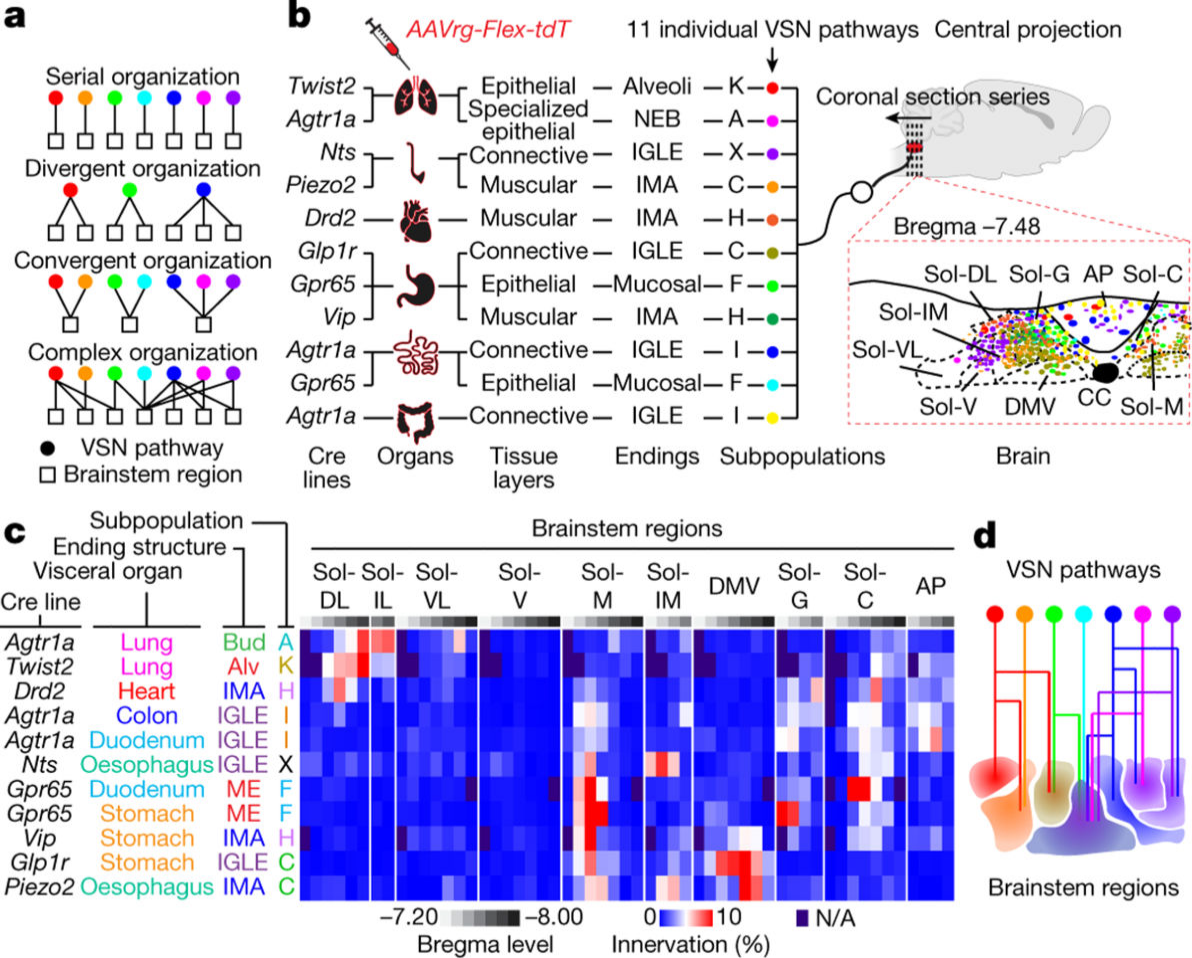
Complex organization of parallel VSN pathways in the brainstem. **a**, Models for central projection patterns of parallel VSN pathways. **b**, Schematic illustration of Projection-seq-guided retrograde tracing of 11 VSN pathways (colour-coded), each with a unique combination of VSN characteristics, via injection of AAVrg-FLEX-tdTomato in the indicated organs and Cre mice. Fluorescence-labelled afferent terminals from different VSN pathways in the brainstem at bregma level −7.48 mm are illustrated. AP, area postrema; CC, central canal; DMV, dorsal motor nucleus of the vagus; Sol-C, commissural NTS; Sol-DL, dorsolateral NTS; Sol-G, gelatinosus NTS; Sol-IM, intermediate NTS; Sol-M, medial NTS Sol-V, ventral NTS; Sol-VL, ventrolateral NTS. Organ illustrations were adapted from BioRender.com. Mouse brain illustration adapted with permission from ref. ^11^. **c**, Innervation density of the 11 VSN pathways, expressed as fluorescence in indicated area/fluorescence in total area, along the rostral–caudal axis of the brainstem, from Bregma −7.2 mm to −8.0 mm (mean; colour-coded; *n* = 3 per pathway). Sol-IL, interlateral NTS. N/A, no signal. **d**, Model for a complex divergent–convergent organization of parallel VSN pathways in the brainstem.

Finally, we asked how this multidimensional coding architecture facilitates the organization of VSN afferents in the brain. Central projection specificity for both organ-labelled and genetically defined VSNs has been extensively described^7,11–13,16,19,26,46,47^. Consistently, VSN central projections labelled using AAVrg-tdTomato from respiratory, cardiovascular and digestive systems were largely segregated, whereas vagal afferents from functionally related organs terminated in more overlapping areas (Extended Data Fig. 13), suggesting that parallel vagal pathways might innervate designated, non-overlapping brainstem regions (Fig. 4a).

**Extended Data Fig. 14 |:**
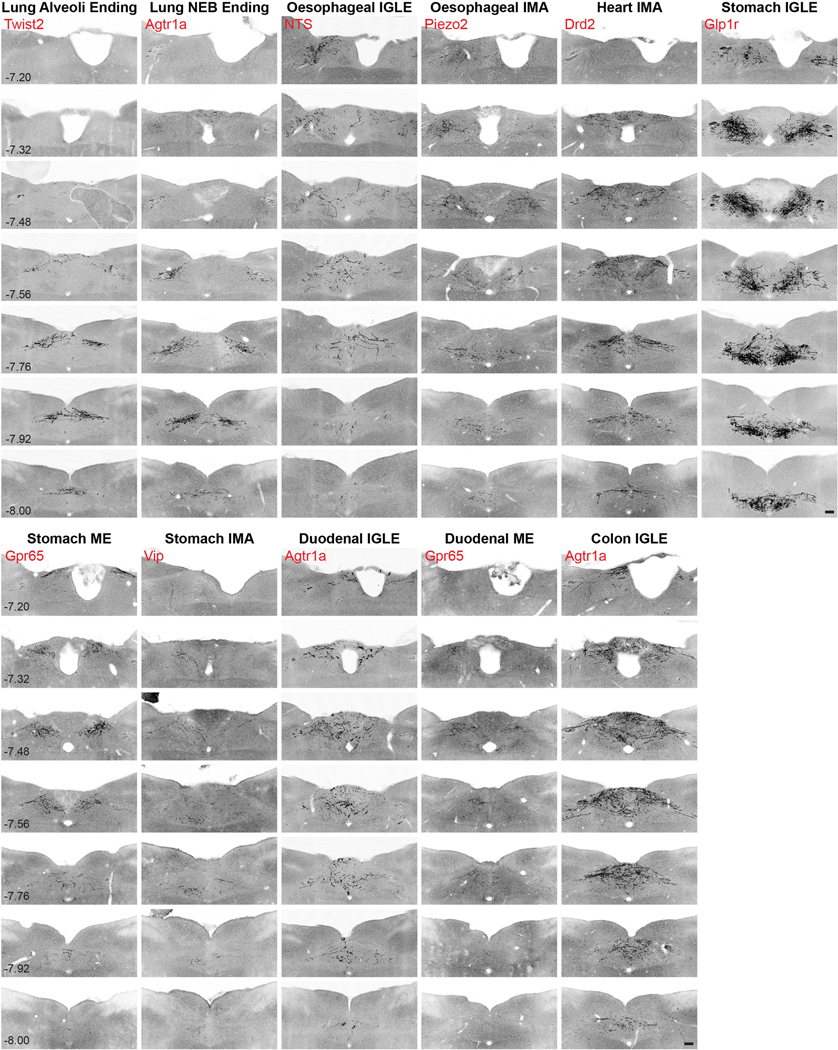
Central projections of 11 individual VSN pathways. Representative brainstem images at indicated Bregma levels along the rostral-caudal axis containing central projections of 11 VSN pathways shown in Fig. 4b. Visceral organ, ending type, and Cre lines used (red) are indicated. Fig. 4b. Visceral organ, ending type, and Cre lines used (red) are indicated. Scale bar: 100 μm.

To test this idea, we performed Projection-seq-guided pathway-specific tracing using organ injection of AAVrg-FLEX-tdTomato in various Cre mouse lines for 11 individual vagal pathways, each representing a unique ‘visceral organ–tissue layer–stimulus modality’combination (Fig. 4b). Notably, pathway-specific tracing did not result in a more distinguishable topographic map in the brainstem (Fig. 4b–d, Extended Data Fig. 14). All 11 of the examined vagal pathways projected to multiple brainstem regions, and vice versa, most brainstem regions received convergent inputs from many VSN pathways (Fig. 4c). The projection of VSNs to different brainstem regions depends on different features of interoceptive signals. Visceral organ is a main factor that determines the projection of VSNs to the lateral versus the medial nucleus of the solitary tract (NTS): all targeted VSN pathways from the lung and heart projected to the dorsolateral NTS, which integrates cardiopulmonary inputs^47^, whereas the medial NTS received various interoceptive signals from all gastrointestinal organs with indistinguishable patterns, therefore functioning as a general gastrointestinal centre. Similarly, stimulus modality drives a marked segregation of gut VSNs in some brainstem regions: mechanosensitive C- and H-VSNs projected extensively to the DMV for real-time regulation of gastrointestinal motility, whereas F- and I-VSNs—both sensing intestinal luminal contents—heavily innervated the gelatinosus and commissural NTS. Our results thus show that parallel VSN pathways are no longer processed in serial in the brainstem but in a more complex manner of divergence and convergence (Fig. 4d).

## Discussion

**Fig. 5 |:**
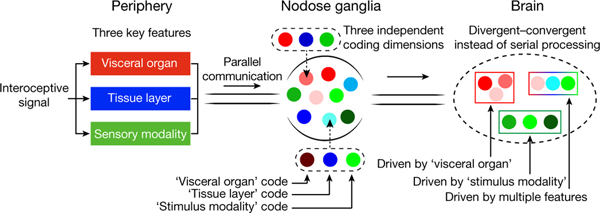
A multidimensional coding architecture of the vagal interoceptive system. Model illustrating the three coding dimensions for three key features of interoceptive signals—visceral organ (red shades), tissue layer (blue shades) and sensory modality (green shades)—in VSNs. This multidimensional coding architecture together specifies many parallel VSN pathways in a combinatorial manner to precisely and effectively present body signals to the brain. Parallel VSN pathways are no longer organized in serial, but in a more complex divergent and convergent manner in the brainstem, based on multiple features of interoceptive signals. The multidimensional coding architecture further facilitates the extensive regrouping of parallel VSN pathways in the brain.

A major challenge in interoception is to communicate an enormous and diverse set of body changes to the brain in an accurate and effective manner. The vagus nerve is a key interoceptive system that surveys various visceral organs. Our study, through a high-throughput large-scale integration of genetic signatures, response patterns and neuronal projections, uncovers a multidimensional coding architecture of the sensory vagus nerve that enables the massively parallel presentation of interoceptive signals in an efficient manner (Fig. 5).

Special coding strategies develop to accommodate different sensory needs. The olfactory system uses combinatorial codes to discriminate numerous odours, and taste receptor cells are organized into labelled lines to distinguish five taste modalities^48–50^. Like gustatory cells, VSNs are grouped into separate and parallel processing streams^12^. However, it is unclear how such organization could satisfy the considerable processing needs. Our results demonstrate that each body region, composed of a unique combination of visceral organ and tissue layer, is surveyed by designated VSNs that are specialized by two independent sets of genes (Fig. 2f). Physically connected organs or tissue layers are generally coded through continuous changes of gene expression in VSNs, which further increases the coding efficiency. As a whole, this strategy ensures a precise coding of the body’s internal space with minimal genes. Our vCatFISH study suggests that VSNs form broad functional units, each with a unique response pattern that recognizes a specific stimulus modality. Although future studies are required to reveal the exact sensory inputs for most VSN subpopulations, our work shows that through this mechanism, VSNs can sense categorized inputs with minimal specialized sensory mechanisms for visceral organs or tissue layers. Together, the vagal interoceptive system codes essential features of interoceptive signals in independent dimensions and uses a combinatorial strategy to effectively expand diversity. This multidimensional coding architecture not only enables the generation of many parallel VSN processing streams but also facilitates the complex projection of vagal afferents in the brain. Thus, the coding mechanism that we have identified here is a good demonstration of the ‘efficient sensory coding theory’^37,38^ in the interoceptive system.

Abnormal presentation of interoceptive signals often leads to global dysfunction, which causes multiple psychological and physiological disorders^4^. Looking forward, the presented integrative approach will provide an effective blueprint for systematically unravelling the molecular and functional architecture of the interoceptive system, and will inspire innovative therapies for disease treatment.

### Online content

Any methods, additional references, Nature Research reporting summaries, source data, extended data, supplementary information, acknowledgements, peer review information; details of author contributions and competing interests; and statements of data and code availability are available at https://doi.org/10.1038/s41586-022-04515-5.

## Methods

### Mice

All animal husbandry and procedures were performed in compliance with Yale University’s Institutional Animal Care and Use Committee and National Institute of Health (NIH) guidelines. All mice were age-and gender-matched adults (older than 8 weeks) and no differences between sexes were observed.

### Mouse lines

Wild-type C57BL/6J (000664), *Glp1r-ires-Cre* (029283), *Gpr65-ires-Cre* (029282), *Npy2r-ires-Cre* (029285), *P2ry1-ires-Cre* (029284), *Agtr1a-Cre* (030553), *Calb2-ires-Cre* (010774), *Nts-Cre* (017525), *Vglut2-ires-Cre* (016963), *Sst-ires-Cre* (013044), *Twist2-Cre* (008712), *Vip-ires-Cre* (010908), *Piezo2-GFP-ires-Cre* (027719), *Trpv1-Cre* (017769), *Pvalb-Cre* (017320), *Vglut1-ires2-Cre* (023527), *Chat-ires-Cre* (031661), *lox-ChR2* (024109), *lox-tdTomato* (007914), and *Snap25–2A-GCaMP6s-D* (025111) were from the Jackson Laboratory. *Drd2-Cre* (032108-UCD) mice were from the Mutant Mouse Resource and Research Center (MMRRC). *lox-L10-GFP* mice were described before^11,12^.

### Generation of Projection-seq AAVs

UPB sequences were cloned from coding sequences of hChR2 (Addgene, plasmid 28017), hM3Dq (Addgene, plasmid 50474) and hM4Di (Addgene, plasmid 50475) and inserted right before the SV40 poly (A) of AAVrg-CAG-tdTomato-WPRE-SV40 (Addgene, plasmid 59462) using the In-Fusion HD Cloning Kit (Takara, 638909). Projection-seq AAVs (at titre > 10^12^–10^13^ viral genomes per ml) were generated at the UNC Vector Core. Plasmids have been deposited to Addgene.

#### UPB sequences (5′–3′):

##### UPB-oesophagus (UPB5, Addgene plasmid 180783):

ACAGCACCATCCTCAACTCCACCAAGTTACCCTCATCGGACAACCTG CAGGTGCCTGAGGAGGAGCTGGGGATGGTGGACTTGGAGAGGAAAGCC GACAAGCTGCAGGCCCAGAAGAGCGTGGACGATGGAGGCAGTTTTCC AAAAAGCTTCTCCAAGCTTCCCATCCAGCTAGAGTCAGCCGTGGACAC AGCTAAGACTTCTGACGTCAACTCCTCAGTGGGTAAGAGCACGGCCA CTCTACCTCTGTCCTTCAAGGAAGCCACTCTGGCCAAGAGGTTTGCTC TGAAGACCAGAAGTCAGATCACTAAGCGGA.

##### UPB-stomach (UPB1, Addgene plasmid 180784):

ATGGACTATGGCGGCGCTTTGTCTGCCGTCGGACGCGAACTTTTGTT CGTTACTAATCCTGTGGTGGTGAACGGGTCCGTCCTGGTCCCTGAGGAT CAATGTTACTGTGCCGGATGGATTGAATCTCGCGGCACGAACGGCGC TCAGACCGCGTCAAATGTCCTGCAGTGGCTTGCAGCAGGATTCAGCAT TTTGCTGCTGATGTTCTATGCCTACCAAACCTGGAAATCTACATGCGG CTGGGAGGAGATCTATGTGTGCGCCATTGAAATGGTTAAGGTGATTCTC GAGTTCTTTTTTGAGTTTAAGAATCCCTCTATGCTCTACCTT.

##### UPB-duodenum (UPB6, Addgene plasmid 180785):

AATGGCAGCTCGGGCAATCAGTCCGTGCGCCTGGTCACGTCATCAT CCCACAATCGCTATGAGACGGTGGAAATGGTCTTCATTGCCACAGTGA CAGGCTCCCTGAGCCTGGTGACTGTCGTGGGCAACATCCTGGTGATGC TGTCCATCAAGGTCAACAGGCAGCTGCAGACAGTCAACAACTACTTCCT CTTCAGCCTGGCGTGTGCTGATCTCATCATAGGCGCCTTCTCCATGAAC CTCTACACCGTGTACATCATCAAGGGCTACTGGCCCCTGGGCGCCGTG GTCTGCGACCTGTGGCTGGCCCTGGACTGCGTGGTGAGCAACGCCTC CGTCATGAACCTTCTCATCATCAGCTTTGACCGCTACTTCTGCGTCA.

##### UPB-colon (UPB4, Addgene plasmid 180786):

ATCGATGGGCCTTAGGGAACTTGGCCTGTGACCTCTGGCTTGCCA TTGACTGCGTAGCCAGCAATGCCTCTGTTATGAATCTTCTGGTCATCA GCTTTGACAGATACTTTTCCATCACGAGGCCGCTCACGTACCGAGCCAA ACGAACAACAAAGAGAGCCGGTGTGATGATCGGTCTGGCTTGGGTCAT CTCCTTTGTCCTTTGGGCTCCTGCCATCTTGTTCTGGCAATACTTTGTT GGAAAGAGAACTGTGCCTCCGGGAGAGTGCTTCATTCAGTTCCTCAGT GAGCCCACCATTACTTTTGGCACAGCCATCGCTGGTT.

##### UPB-pancreas (UPB7, Addgene plasmid 180787):

AAGATGGCAGGCCTCATGATTGCTGCTGCCTGGGTACTGTCCTTCGT GCTCTGGGCGCCTGCCATCTTGTTCTGGCAGTTTGTGGTGGGTAAGC GGACGGTGCCCGACAACCAGTGCTTCATCCAGTTCCTGTCCAACCCA GCAGTGACCTTTGGCACAGCCATTGCTGGCTTCTACCTGCCTGTGGT CATCATGACGGTGCTGTACATCCACATCTCCCTGGCCAGTCGCAGCCG AGTCCACAAGCACCGGCCCGAGGGCCCGAAGGAGAAGAAAGCCAAGA CGCTGGCCTTCCTCAAGAGCCCACTAATGAAGCAGA.

##### UPB-lung (UPB2, Addgene plasmid 180788):

ACAGGACACCGGGTGCAGTGGCTGCGCTATGCAGAGTGGCTGCTCA CTTGTCCTGTCATCCTTATCCGCCTGAGCAACCTCACCGGCCTGAGCA ACGACTACAGCAGGAGAACCATGGGACTCCTTGTCTCAGACATCGGGA CTATCGTGTGGGGGGCTACCAGCGCCATGGCAACCGGCTATGTTAAA GTCATCTTCTTTTGTCTTGGATTGTGCTATGGCGCGAACACATTTTTTC ACGCCGCCAAAGCATATATCGAGGGTTATCATACTGTGCCA.

##### UPB-heart (UPB8, Addgene plasmid 180789):

AGGACACTTCCAATGAGTCCAGCTCAGGCAGTGCCACCCAGAACACC AAGGAACGCCCAGCCACAGAGCTGTCCACCACAGAGGCCACCACGCC CGCCATGCCCGCCCCTCCCCTGCAGCCGCGGGCCCTCAACCCAGCC TCCAGATGGTCCAAGATCCAGATTGTGACGAAGCAGACAGGCAATGAG TGTGTGACAGCCATTGAGATTGTGCCTGCCACGCCGGCTGGCATGCGC CCTGCGGCCAACGTGGCCCGCAAGTTCGCCAGCATCGCTCGCAACCA GGTGCGCAAGAAGCGGCAGATGGCGGCCCGGGAGCGCAAAGTGACAC GAACGATCTTTGCCATTCTGCTGGCCTTCATCCT.

### AAV infection of visceral organs

Retrograde adeno-associated virus (AAVrg)^51^ represents a powerful genetic tool in VSN studies^52–54^. AAVrg-tdTomato and AAVrg-GFP were purchased from the UNC vector core. AAVrg-FLEX-tdTomato (28306-AAVrg), AAVrg-CAG-FLEX-rc [ Jaws-KGC-GFP-ER2] (84445-AAVrg) and pAAVrg-hSyn-Con/Fon hChR2(H134R)-EYFP (55645-AAVrg) were purchased from Addgene. All viruses contained 10^12^–10^13^ viral genomes per ml, and 0.05% Fast Green FCF (Sigma-Aldrich, F7252–5G) was occasionally used to facilitate visualization.

In all surgeries, mice were anaesthetized with 1–2% isoflurane on a heating pad, followed by subcutaneous injections of meloxicam (5 mg kg^−1^) and buprenorphine (1.5 mg kg^−1^). Lung: virus (5 μl diluted with 75 μl of saline) was injected through a tracheal catheter into the lung using a Hamilton syringe. Heart: the mouse was ventilated (tidal volume 0.21 ml, respiration rate 110 breaths per minute) using a mouse ventilator (SAR-1000, CWE) via an intubated angiocatheter. The heart was gently exposed via thoracotomy. Virus (5 μl) was injected intramurally (20 nl per second) using a Nanoject III injector at multiple sites covering most of the area of the heart. Stomach, duodenum, colon and pancreas: the target organ was gently exposed via an abdominal incision. Virus was injected intramurally at multiple sites covering most of the area (stomach: 5 μl; all subregions in both dorsal and ventral sides; duodenum: 2.5 μl, a length of 1.5 cm from the pyloric sphincter; transverse colon: 2.5 μl, a length of 1.5 cm; pancreas: 5 μl). Oesophagus: the cervical oesophagus underneath the trachea was surgically exposed via a neck incision. The abdominal oesophagus and the oesophageal sphincter were gently exposed via an abdominal incision. Virus was injected into oesophageal muscularis and serosa layers (cervical: 2.5 μl; abdominal: 1 μl, between the diaphragm and the oesophageal sphincter).

To visualize and quantify the anatomical location of VSNs innervating various visceral organs in the nodose ganglion (Extended Data Fig. 1a–c), the following AAVrgs were injected into visceral organs in wild-type mice for Extended Data Fig. 1a–c: heart (tdTomato)/lung (GFP), heart (tdTomato)/stomach (GFP), heart (tdTomato)/oesophagus (GFP), pancreas (tdTomato)/stomach (GFP), heart (tdTomato)/duodenum (GFP), and colon (tdTomato). To compare infection efficiencies of AAVrg and the conventional neural tracer cholera toxin B subunit (CTb), AAVrg-tdTomato (2.5 μl) and Alexa Fluor 647-conjugated CTb (1.0 mg ml^−1^, 2.5 μl, Thermo Fisher Scientific, C34778) were mixed and co-injected into the stomach in wild-type mice (Extended Data Fig. 1d). AAVrg and CTb labelled partially overlapping VSNs with similar efficiencies, raising the possibility that AAVrg might have some preferential tropism for specific VSN populations over CTb. However, it is worth noting that co-infection of VSNs with two similar AAVs (AAV9-FLEX-tdTomato, AAV9-GFP) via nodose injection also resulted in similar partially overlapping labelling^11^, suggesting that this likelihood is low. This possibility is further reduced by the extensive validation of Projection-seq results using anterograde tracing in 14 Cre mouse lines as described in Extended Data Figs. 4–8.

To verify the level of coverage of injected virus within the organ (Extended Data Fig. 1e–q, s–u), AAVrg-tdTomato or Fast Green FCF (5% w/v, same volume as AAVrg) were injected into wild-type mice as described above. Organs were processed as described in ‘Histology and immunochemistry’. Injected dye or virus were able to cover most subregions and virus-infected cells were observed in most tissue layers including the pulmonary alveoli, stomach and intestinal villi, and the entire myocardial layer of the heart ventricles. We also noticed that the atria and bronchial walls were not well covered. To examine whether AAVrgs could label vagal motor neurons in the DMV, AAVrg-FLEX-tdTomato (2.5 μl) and AAVrg-GFP (2.5 μl) were mixed and co-injected into the stomach of *Chat-ires-Cre* mice (Extended Data Fig. 1r). To examine the degree of viral spread (Extended Data Fig. 1v, w), AAVrg-tdTomato and AAVrg-GFP were separately injected into indicated organs in the same mice. To determine the infection efficiency of Projection-seq AAVs (Extended Data Fig. 2a, b), Projection-seq AAVs and AAVrg-GFP were injected into the stomach. For Projection-seq analysis (Fig. 1), Projection-seq AAVs with UPB-lung, UPB-heart, UPB-oesophagus, UPB-stomach, UPB-duodenum, UPB-colon and UPB-pancreas were injected into corresponding organs. The heart, cervical oesophagus and lung were sequentially injected on the first day, and abdominal oesophagus, stomach, duodenum, pancreas and colon were sequentially injected into the same mice on the second day. Approximately 30% of VSNs were labelled from the seven organs (Extended Data Fig. 2c). For RNAscope HiPlex assays of retrogradely labelled VSNs (Extended Data Fig. 2p, q), AAVrgs encoding reversed transcripts of fluorescent proteins, including AAVrg-FLEX-tdTomato (heart, stomach, duodenum), AAVrg-CAG-FLEX-rc [Jaws-KGC-GFP-ER2] (lung, oesophagus, pancreas) and AAVrg-hSyn Con/Fon hChR2(H134R)-EYFP (colon) were used to avoid potential contamination of RNAscope results from virus-introduced fluorescent signals.

For tracing the central targets of diverse vagal pathways, AAVrg-tdTomato was injected into visceral organs of wild-type mice (Extended Data Fig. 13), and AAVrg-FLEX-tdTomato was injected into visceral organs of indicated Cre lines (Fig. 4, Extended Data Fig. 14). Vagal ganglia were collected seven days after surgery and brains were collected two to three weeks after surgery.

### PCR analysis of VSNs

Vagal ganglia were collected from control mice or mice with stomach injection of AAVrg-tdTomato-UPB2. RNA was extracted using the Monarch Total RNA Miniprep Kit (NEB, T2010S) and reverse-transcribed into cDNA using the SuperScript IV First-Strand Synthesis System (Thermo Fisher Scientific, 18091050). Primer sets used (Extended Data Fig. 2b):

UPB2 (5′–3′): CATCGATACCGTCGACACAGGACACCGGGTGCAGTG (forward); CTGCTCGAAGCGGCCGCTGGCACAGTATGATAACCCTCG (reverse). UPB4 (5′–3′): CATCGATACCGTCGACATCGATGGGCCTTAGG GAAC (forward); TGCTCGAAGCGGCCGCAACCAGCGATGGCTGTGCC (reverse).

### scRNA-seq and Projection-seq of VSNs innervating various visceral organs

#### Neuron isolation and sequencing.

For control scRNA-seq, vagal ganglia (left and right) were collected from 40 age- and gender-matched C57BL/6J wild-type mice (10 mice per sample) and VSNs were acutely isolated and enriched using previously described methods^11,12,17^. Approximately 5,000–10,000 VSNs were loaded in each channel of the 10X microfluidic device to target 3,000–6,000 cells as an output of one sample. Single-cell cDNA libraries were prepared at the Yale Center for Genomic Analysis (YCGA) and sequenced using an Illumina NovaSeq S4 sequencer at 150–300 million reads to achieve a fine sequencing depth of 30,000–50,000 reads per cell. For Projection-seq, 30 age- and gender-matched wild-type mice (divided into four samples) were first injected with different Projection-seq AAVs into thoracic and abdominal organs (details in ‘AAV infection of visceral organs’). Vagal ganglia were collected seven days later. Of the examined VSNs, 29.5% (102/346) were tdTomato^+^. VSNs were sequenced as mentioned above.

#### Bioinformatic processing.

Transcriptomic data were aligned to the mm10 mouse genome reference (control scRNA-seq) or a custom mouse genome reference with additional sequence information of UPBs (Projection-seq) using the Cell Ranger software v.3.0.2 (10X Genomics). The following quality control metrics were applied to filter low-quality cells in scRNA-seq: number of genes per cell > 500; number of genes per cell < 8,000; percentage of mitochondria genes < 10%. A total of 56,575 cells were sequenced (31,182 control scRNA-seq samples: 7,842, 7,580, 7,939, 7,821; 25,393 Projection-seq samples: 6,403, 6,381, 5,803, 6,806). Control scRNA-seq and Projection-seq data were then integrated and processed using the R package Seurat v.3^55^, and 42 cell clusters identified using the top 30 principal components (PCs) were visualized using UMAP^56^ (Extended Data Fig. 2f). A total of 32,558 neurons selected from 25 *Slc17a6*^+^ clusters (16,267 control scRNA-seq samples: 4,961, 3,894, 4,288, 3,124; 16,291 Projection-seq samples: 3,863, 3,431, 4,449, 4,548) were re-clustered into 67 populations with the top 100 PCs (Extended Data Fig. 2g, top). A total of 27,800 placode-derived neurons selected from 52 *Phox2b*^+^ clusters (13,210 control scRNA-seq samples: 3,835, 3,091, 3,452, 2,832; 14,590 Projection-seq samples: 3,443, 2,973, 4,019, 4,155) were re-clustered into 52 clusters with the top 100 PCs and visualized with UMAP separately (Fig.1b for Projection-seq; Extended Data Fig. 2g, bottom for control scRNA-seq) or together (Extended Data Fig. 2h). DEGs for the 52 clusters were identified using the Wilcoxon rank-sum test implemented in Seurat from the Projection-seq dataset (Extended Data Fig. 2d). VSN clusters were then manually grouped into 12 subpopulations on the basis of expression of DEGs and their locations on the UMAP plot. E-VSNs selectively express markers for damaged sensory neurons, such as *Sprr1a* and *Ecel1*^16,57,58^ (Extended Data Fig. 3a). The percentage of E-VSNs increased after the Projection-seq process (control: *n* = 627, 4.7%; Projection-seq: *n* = 1,528, 10.5%), and consistently more *Sprr1a*^+^ VSNs were observed after AAVrg injection (Extended Data Fig. 3b–d), demonstrating that neurons damaged during both cell dissociation and Projection-seq procedures can be easily identified. Thus, E-VSNs were removed from further analysis.

#### Projection-seq analysis.

Further analysis was performed for the Projection-seq dataset. A total of 42 out of 1,701 *Prdm12*^+^ (2.5%) neural-crest-derived jugular VSNs and 4,791 out of 14,590 (32.8%) *Phox2b*^+^ placode-derived nodose VSNs^18,59^ were recognized as UPB-positive (expression level > 0.8), suggesting that VSNs retrogradely labelled from the seven major visceral organs in our study mainly originate from the nodose but not the jugular ganglia. We therefore focused on nodose VSNs. After removing E-VSNs, 3,539 out of 4,609 UPB-marked nodose VSNs (76.8%) expressed a single barcode (lung-UPB, 855; oesophagus-UPB, 595; heart-UPB, 177; stomach-UPB, 1,166; duodenum-UPB, 110; pancreas-UPB, 356; colon-UPB, 280), and 740 out of 4,609 (16.1%) VSNs expressed were marked by two UPBs. A correlation matrix (Extended Data Fig. 2e) was calculated on the basis of the numbers of single-UPB and dual-UPB marked VSNs across the seven examined visceral organs and plotted using the R pheatmap package. Correlation analysis indicates that the two UPBs were predominantly from physically adjacent rather than random organs (Extended Data Fig. 2e). This observation was consistent with retrograde tracing results (Extended Data Fig. 1b, v, w), demonstrating that (1) UPB leakage was minimal during sample preparation; and (2) dual-labelled VSNs innervate regions close to both organs. Single UPB-labelled VSNs were designated as organ-specific VSNs, VSNs dual labelled with UPB-stomach and UPB-oesophagus were designated as oesophageal-sphincter projecting neurons and VSNs dual labelled with UPB-stomach and UPB-duodenum were designated as pyloric-sphincter projecting neurons. To examine VSNs that innervate different physiological systems, VSNs singly marked by oesophagus, stomach, duodenum and colon-UPBs were combined as gut VSNs (Fig. 1c, d). A 3D UMAP plot (Fig. 1c, bottom) was generated using a previously published method^60^ (https://github.com/Dragonmasterx87/Interactive-3D-Plotting-in-Seurat-3.0.0 ). DEGs for organ-specific VSNs, or between thoracic (combination of oesophagus, heart and lung-UPB labelled) and abdominal (combination of stomach, duodenum, colon and pancreas-UPB labelled) VSNs were identified using the Wilcoxon rank-sum test (Extended Data Fig. 3e). Mouse transcription factors were identified (Fig. 1d, Extended Data Fig. 3g) by comparing DEGs with the AnimalTFDB 3.0 mouse database^61^ (http://bioinfo.life.hust.edu.cn/AnimalTFDB/#!/). For example, *Pou4f1*, which is essential for DRG neuron specification^32^, is preferentially expressed in lung VSNs (Extended Data Fig. 3g). Regulatory networks in UPB-labelled VSNs (Extended Data Fig. 3h) showing the expression of *Pou4f1* downstream regulators including *Runx1* and *Isl2* were predicted by IPA analysis (Qiagen) using the Upstream Analysis (Upstream Regulators) module. For cell–cell interaction analysis between organ-innervating VSNs and various organ cell types (Extended Data Fig. 3i), UPB^+^ VSNs labelled from the indicated organ were first extracted from the Projection-seq data and then integrated with a previously published scRNA-seq dataset containing various cell types from that corresponding organ using the R package Seurat v.3^55^. The following datasets were used: heart^62^ (CM, cardiomyocyte; EDC, endothelial cell; EP, epicardial cell; FB, fibroblast), lung^63^ (ATI, alveolar epithelial type I cell; ATII, alveolar epithelial type II cell; B, B cell; C&S, ciliated and secretory cell; DC, dendritic cell; EDC, endothelial cell; FB, fibroblast; MO, monocyte; Mac, macrophage; NK, natural killer cell; Neutro, neurophil; Peri, pericyte; T, T cell), colon^64^ (Endo, endothelial cell; Immu, immune cell; EN, enteric neuron; Glia, glial cell; Entero, enteroendocrine cell; Mus/Fb, muscle cell and fibroblast), duodenum^65^ (duodenal enteric neuron subtypes: EXMN, excitatory motor neuron; INMN, inhibitory motor neuron; IN, inter neuron; IPAN, intrinsic primary afferent neuron) and pancreas^60^ (ISL, islet cell; ACI, acinar cell; DUCT, duct cell; MES, mesenchymal cell; IMVS, immune and vascular cell). Cell–cell interactions between organ-innervating UPB^+^ VSNs and indicated organ cell types (or duodenal enteric neuron subtypes) were then analysed using the CellPhoneDB^66^ (https://github.com/Teichlab/cellphonedb,v.2.0.0 ) Python package. GO pathway analyses of DEGs in heart, lung, gut and pancreas VSNs, and along the tissue trajectory (Extended Data Fig. 8s), were performed using the Gene Ontology Resource GO Enrichment Analysis tool^67–69^ (http://geneontology.org).

#### Calculation of trajectory score.

Distances from the nodose ganglia to the beginning and the end of various organs were measured and normalized to the body length from the neck to the rectum (Fig. 1e–g). The mean distances were calculated as Position_organ_ (lung: 0.241 ± 0.005; heart: 0.280 ± 0.005; oesophagus: 0.228 ± 0.005; stomach: 0.469 ± 0.006; duodenum: 0.567 ± 0.006; transverse colon: 0.589 ± 0.005; pancreas: 0.545 ± 0.007; *n* = 4 mice). The organ position score for VSN clusters (Fig. 1e), indicating their target preference along the body’s rostral–caudal axis, was calculated as the weighted average of Position_organ_ using the percentage of organ-specific VSNs in the cluster (*P*_organ-cluster_ = Number of UPB_organ_^+^ VSNs in the target cluster/Number of all UPB_organ_^+^ VSNs) as the weight value for each organ, expressed as Σ(*P*_organ-cluster_ × Position_organ_)/Σ*P*_organ-cluster_. The organ trajectory score of an organ-specific VSN (Fig. 1f, g) was measured as its projection position along the organ trajectory on the UMAP plot (as shown in Fig. 1e). Tissue layer trajectory was identified using Slingshot^70^, and DEGs along this trajectory were discovered using tradeSeq^71^. Tissue layer trajectory scores of DEG^+^ VSNs were measured as their relative positions along the tissue layer trajectory by Slingslot using the pseudotime function. On the basis of the inner–outer position, each tissue type was given an index score (mucosa or inner epithelium, 0; muscle, 1; connective tissue, 2), and the tissue layer index for DEG^+^ VSNs (Fig. 2a–c) in each organ was calculated based on the percentage of DEG^+^ VSN endings in the target tissue layer and the tissue layer index across multiple tissue layers as Σ((Number of DEG^+^ VSN endings in the target tissue layer/ Number of DEG^+^ VSN endings in all tissue layers) × Index_tissue_) (Fig. 2a–c).

### AAV infection of vagal ganglia

Vagal ganglia injection was performed as previously described^11,12^. In brief, mice were anaesthetized with 1–2% isoflurane and maintained on a heating pad. Both left and right vagal ganglia were surgically exposed. A virus mix containing a 1:1 dilution of AAV9-FLEX-tdTomato and AAV5-CAG-GFP with 0.05% (w/v) Fast Green FCF was injected (160 nl for each side, 20 nl per second) using a Nanoject III injector (Drummond). Mice were euthanized four weeks after surgery for tissue collection (see ‘Histology and immunochemistry’).

### RNAscope HiPlex assay

RNAscope HiPlex assays were performed following the manufacturer’s protocol (Advanced Cell Diagnostics). Vagal ganglia were acutely dissected and freshly frozen in cryo-embedding medium (OCT). Cryosections (10 μm for vCatFISH; 14 μm for others) were cut using a cryostat (Thermo Fisher Scientific), mounted onto Superfrost Plus slides (Thermo Fisher Scientific), and stored at −80 °C until use. Slides were immediately immersed into fresh 4% paraformaldehyde (PFA) in RNase-free PBS for 60 min at room temperature, followed by dehydration with 50%, 70% and 100% ethanol. Samples were then digested with protease IV for 30 min at room temperature. After hybridization with designed probes (Supplementary Table 1) for 2 h at 40 °C, the sections were treated with HiPlex Amp 1–3, and then HiPlex Fluoro T1–T3, before counterstaining and mounting. The slides were imaged using a Leica SP8 confocal microscope equipped with a motorized stage, a PMT detector, a HyD SP detector, four laser lines (405 nm, 488 nm, 552 nm and 638 nm) and a 20× objective (HC PL APO 20×/0.75 CS2). Imaging loci were exactly recorded in the LAS X software in the first group for image registration, and then applied for imaging the same sections for the following groups. After each group, the fluorophores were cleaved by 10% cleaving solution (ACD, 324130). The sections were then hybridized with HiPlex Fluoro T4–T6 in group 2, HiPlex Fluoro T7–T9 in group 3 and HiPlex Fluoro T10–T12 in group 4. In total, 12 genes were detected on a single section. The following changes were made for vCatFISH: (1) slides were not mounted after HiPlex Fluoro and instead imaged in 4× SSC with a 16× immersion objective (HC FLUOTAR L 16×/0.8 IMM motCORR VISIR); (2) after the first-round imaging (4 groups, 12 probes), probes were removed using the HiPlexUp reagent, and sections were hybridized with another 12 probes (2 h, 40 °C) for the second-round analysis. In total, 22 genes were analysed (see ‘vCatFISH analysis’).

### Histology and immunochemistry

Mice were anaesthetized with urethane (1.5 g per kg) and transcardially perfused with 15 ml cold PBS (pH 7.4) containing 10 U ml^−1^ heparin (Sigma-Aldrich, H4784), followed by 25 ml cold 4% PFA. Fast Green FCF injected organs (Extended Data Fig. 1e, g, j, s–u) were dissected and imaged under a Stereo Microscope with a digital camera (AmScope). For all others, visceral organs and/or vagal ganglia were dissected, post-fixed in 4% PFA at 4 °C (overnight for visceral organs, 30 min for ganglia), and then kept in cold PBS at 4 °C. Brains were post-fixed (4% PFA, overnight, 4 °C), cryoprotected in 30% sucrose PBS solution for two days at 4 °C, frozen in OCT and then stored at −80 °C until cryosection.

For brain samples, 40-μm cryosections were mounted onto Superfrost Plus slides. Cryosections were washed (3× PBS), permeabilized (0.1% Triton X-100, PBS), blocked (5% normal donkey serum, PBST (PBS, 0.05% Tween-20)) and incubated with primary antibodies (chicken anti-GFP, 1:1,000, Aves Labs; rabbit anti-RFP, 1:1,000, Rockland) diluted in blocking buffer for 2 h at room temperature. Then, the slides were washed (3× PBST), and incubated with fluorophore-conjugated secondary antibodies (Alexa Fluor 647-conjugated AffiniPure donkey anti-chicken IgY, 1:1,000; Alexa Fluor 594-conjugated AffiniPure donkey anti-rabbit IgG(H+L), 1:1,000, Jackson ImmunoResearch) diluted in blocking buffer for 2 h at room temperature. After incubation, the samples were washed (3× PBST), and mounted with Fluoromount-G with DAPI before imaging with the Leica SP8 confocal microscope.

Visceral organs were cleared with the CUBIC method^72^ and stained with the following protocol unless specifically mentioned. In brief, the dissected organ was immersed into 1/2-water-diluted reagent-1 (25 wt% urea, 25 wt% Quadrol, 15 wt% Triton X-100) with shaking at 37 °C for 3–6 h, followed by reagent-1 (R1) with shaking at 37 °C for 7 days. R1 was replaced fresh every two days. Next, the tissue was washed (3× PBS/0.01% NaN_3_), blocked (2% normal donkey serum, 0.1% Triton X-100, PBS/0.01% NaN_3_) and incubated with primary antibodies (chicken anti-GFP, 1:200; rabbit anti-RFP, 1:200) in blocking buffer with shaking for 7 days at room temperature. Samples were then washed (0.1% Triton X-100, PBS/0.01% NaN_3_) and incubated with fluorophore-conjugated secondary antibodies diluted in blocking buffer with shaking for five days at room temperature. After antibody incubation, the samples were washed and immersed in 1/2-PBS-diluted reagent-2 (25 wt% urea, 50 wt% sucrose, 10 wt% triethanolamine) overnight at room temperature, and then reagent-2 (R2) at 37 °C for 2 days. The samples were finally immersed in oil for at least 1 h and flattened to approximately 500 μm in a custom-built imaging chamber and imaged using the Leica SP8 confocal microscope as described above, with a 10× objective (HC PL APO 10×/0.40 CS2, working distance: 2.1 mm) or a 40× objective (HC PL FLUOTAR L 40×/0.60 CORR, working distance: 3.3 mm). Some heart samples were similarly processed but not flattened and imaged with a LaVision Vltramicroscope II light-sheet microscope at the CNNR Imaging Core at Yale University (Extended Data Fig. 5a) or the Leica SP8 confocal microscope with a 16× immersion objective (HC FLUOTAR L 16×/0.8 IMM motCORR VISIR, working distance: 8 mm). Heart slices (1 mm thickness) and gastrointestinal organs to determine viral spread (Extended Data Fig. 1f, v, w) were imaged under a Leica M205FCA Fluorescent Stereo Microscope with a CoolLED pE300 white illumination, GFP and DSR Filter sets, 1× objective (Plan M-series) and a Leica DFC7000 T camera. The pancreas was excluded from anatomical analysis owing to multiple technical challenges (keeping its original structure and distinguishing various tissue layers after clearing, antibody penetration and imaging efficiency).

A modified CUBIC protocol was used for clearing vagal ganglia: samples were cleared in R1 for one day, incubated in primary and secondary antibodies (chicken anti-GFP, 1:1,000; rabbit anti-RFP, 1:1,000; Alexa Fluor 647-conjugated AffiniPure donkey anti-chicken IgY, 1:1,000; Alexa Fluor 594-conjugated AffiniPure donkey anti-rabbit IgG(H+L), 1:1,000) overnight, respectively, and treated with R2 for one day. Cleared ganglia were imaged with the Leica SP8 confocal microscope.

### vCatFISH analysis

#### Surgery, stimulus delivery and imaging.

Gpr65^tdT^-GCaMP6s mice were continuously anaesthetized (1–2% isoflurane/oxygen) during the experiment. A tracheal tube was inserted for air injection. The upper oesophagus and pyloric sphincter were cannulated and flushed with saline multiple times to remove residual food particles. The duodenum (around 0.5 cm below the pyloric sphincter) was cannulated with a bundle of six PE-10 tubing for separate delivery of saline, water and glucose (1 M), Ensure (Ensure Original Vanilla Nutrition Shake), 10× PBS and 150 mM HCl (pH = 0.84). Left vagal ganglia were exposed and immobilized on a stable platform^12^. During calcium imaging, a series of stimuli were delivered to the same mice in the following sequence: (1) lung inflation for 20 s with 600 ml min^−1^ flow (oxygen through the tracheal tube), twice with a 2-min interval; (2) small intestine stretch via fast injection of 600 μl saline through the duodenal cannula; stomach stretch with 100 μl, 300 μl and 600 μl saline through the oesophageal cannula for 30 s (duration precisely controlled by closing or opening of the pyloric cannula); (4) small intestine infusion with 100 μl saline, water, 1 M glucose, Ensure, 10× PBS and 150 mM HCl in sequence with a 3-min interval between infusions. GCamp6s fluorescence was measured from two focal planes 15 μm apart using a two-photon microscope (920 nm excitation, Leica TCS SP8, Mai Tai laser from Spectra-Physics, HyD SP detector). The imaging frequency for each plane was 1.72 s per frame. At the end, electrical stimulation was applied to the vagus nerve and a *z*-stack of the ganglia was taken for both GCaMP and tdTomato (552 nm, one photon excitation) signals for cell registration.

#### RNAscope and registration.

After GCaMP imaging, vagal ganglia were immediately embedded in OCT, frozen in situ, and cut into 10 um cryosections. RNAscope HiPlex assays for the following 22 genes were performed (Fig. 3b, Extended Data Fig. 9): *Trpa1* (R1T1), *Runx3* (R1T2), *Uts2b* (R1T3), *Gabra1* (R1T4), *Slit2* (R1T5), *Kcng1* (R1T6), *Piezo2* (R1T7), *Ddc* (R1T8), *Vip* (R1T9), *Trpv1* (R1T10), *tdTomato* (R1T11), *Gpr65* (R1T12), *Chodl* (R2T1), *Glp1r* (R2T2), *Grm5* (R2T3), *Slc17a7* (R2T4), *P2ry1* (R2T5), *Tmc3* (R2T6), *Car8* (R2T7), *Nts* (R2T8), *Cckar* (R2T9) and *Calca* (R2T11). The following criteria were used to categorize VSN subpopulations (Fig. 3b, Extended Data Fig. 9): A-VSNs: *Runx3^+^* and/or *Piezo2^+^*/*P2ry1^+^*, *Tmc3^−^*; B-VSNs: *Gabra1*^+^; C-VSNs: multiple hits for *Slit2^+^*, *Piezo2^+^*, *Ddc^+^*, *Tmc3^+^*, *Trpv1*^−^, *P2ry1^−^*. D-VSNs: *Tmc3^+^*, *Trpv1^−^*; F-VSNs: *Gpr65^+^*; G-VSNs: *Trpv1^+^*, multiple hits for *Uts2b^+^*, *Vip^+^*, *Glp1r^+^*, *Cckar^+^*; H-VSNs: *Trpv1^+^*, *Tmc3^−^*, *Trpa1^+^*; I-VSNs: multiple hits for *Tmc3^+^*, *Car8^+^*, *Cckar^+^*, *Piezo2^−^*. J -VSNs: *Trpv1*^+^, *Calca^+^* and some *P2ry1^+^*, *Piezo2^−^*; K-VSNs: *Trpa1^+^*, *Kcng1^+^*, *Trpv1^+^*, *Calca^+^*; L-VSNs: *P2ry1^+^*, *Trpv1^−^*. tdTomato^+^ neurons that serve as geographic landmarks for cell registration were registered first between in vivo GCaMP images and RNAscope images (Extended Data Fig. 9e). Coordinates of registered tdTomato^+^ cells were then used to calculate the transformation matrix between in vivo 3D images and RNAscope sections. We reason that tdTomato^+^ cells from a given RNAscope section should also be located in the same plane in the transformed in vivo image stack, therefore we used a plane correction script simulating a virtual plane with minimum total projection distances for tdTomato^+^ cells from multiple RNAscope sections. The transformation matrix was then applied to the in vivo image stack (3D extension/plug-in in LASX software) to generate successive in vivo imaging planes that resemble RNAscope sections. tdTomato^−^ neurons were then registered on the basis of their relative distance and depth to their neighbouring tdTomato^+^ cells (Extended Data Fig. 9e–g). Registration for each sample was performed by at least two people independently. In total, 57.5% (349/607, 6 mice) of responsive VSNs were unambiguously registered, which is comparable to a similar approach developed in the trigeminal ganglion^73^. Cells that were not successfully registered were removed from further analysis.

#### Analysis of neural activity.

Regions of interest were manually extracted from GCaMP images. The stimulus induction frame was set as 0 unless specifically mentioned. Baseline signal (*F*) was defined as the average GCaMP6s fluorescence over a 10-frame period (17.2 s, frame −20 to frame −10) before stimulus induction and neuronal activity was calculated as Δ*F*/*F*. Cells were coded as responsive to a given stimulus if the maximum GCaMP6s fluorescence was more than 100% above baseline during stimulus period (lung inflation and stomach stretch: between stretch on and off; intestine stretch: within 40 frames/68.8 s after injection; intestine infusion: within 90 frames/154.8 s after infusion). Peak response was identified as the maximum Δ*F*/*F* within the stimulus period. To compare adaptation rates in lung stretch-sensitive VSNs, GCaMP6s traces were aligned at activation frame (Extended Data Fig. 11c, arrow, set as 0), and the activation duration was calculated as the number of frames between the prior and post peak frames at which VSN activity (Δ*F*/*F*) reached 10% of the peak response (Extended Data Fig. 11d). For intestine stretch, activation frame was defined as the prior peak frame at which VSN activity (Δ*F*/*F*) reached 10% of peak response.

### Projection-seq-guided anterograde tracing in visceral organs

The percentage of gene^+^ VSNs targeted in various Cre lines in individual clusters (Extended Data Fig. 4c) was calculated as the number of gene^+^ VSNs normalized by the number of all VSNs in each cluster using the control scRNA-seq dataset. Fold enrichment of gene^+^ VSNs in each cluster (Extended Data Fig. 4d) was calculated as the percentage of gene^+^ VSNs in the target cluster normalized by the overall percentage of gene^+^ VSNs in all clusters.

#### Identification of enriched clusters and DEGs.

Primary oesophagus, stomach, duodenum, colon, heart and lung VSN clusters were identified as clusters containing more than 4% of corresponding UPB single-labelled VSNs (Extended Data Figs. 5c, 6b, 7c, 8a, e, k, r), with the following additions: G2, G5-duodenum VSNs (both 3.64%) and I4-heart VSNs (3.95%). VSN clusters enriched for stomach regions 4, 6–8 (Extended Data Fig. 7e, h) were defined using the following criteria: (1) the cluster contains more than 4% of dual-UPB (region 4: oesophagus/ stomach; region 6: stomach/pancreas; region 7: stomach/colon;region 8: stomach/duodenum, respectively) labelled VSNs; and (2) in the cluster, the percentage of dual-UPB labelled VSNs is at least 5% higher than both the percentage of stomach-UPB labelled VSNs and the percentage of the other UPB (oesophagus, pancreas, colon, duodenum, respectively) labelled VSNs. VSN clusters enriched for stomach region 5 (fundus, S-only, Extended Data Fig. 7h) were defined as: (1) the cluster contains more than 4% of stomach-UPB labelled VSNs; and (2) in the cluster, the percentage of stomach-UPB labelled VSNs is at least 5% higher than the percentages of stomach/oesophagus, stomach/ pancreas, stomach/colon and stomach/duodenum dual-UPB labelled VSNs. Among the eight primary duodenum VSN clusters (F1, F4, G2, G5, H2, H3, I5 and J3), three (F1, H2, J3) were more enriched in stomach/duodenum dual-labelled VSNs, suggesting that they preferentially project to the pyloric sphincter over the duodenum. Therefore, the other five duodenum VSN clusters were focused on for DEG analysis (Extended Data Fig. 8a). Fractions of DEGs (Extended Data Figs. 5e, 6c, 7f, 8b, f, l) were calculated as the number of DEG^+^ VSNs in the indicated cluster divided by the number of DEG^+^ VSNs in all enriched clusters indicated in the panel. Percentages of gene^+^ VSNs in identified enriched clusters (Extended Data Figs. 5d, 6d, 8h) were calculated as the number of gene^+^ VSNs divided by the total number of VSNs in that cluster.

#### Histology analysis.

Four types of gut VSN ending types, three types of heart VSN ending types and five types of lung VSN ending types were classified on the basis of their morphologies and locations using whole-mount preparations in Vglut2^tdT^ mice (Fig. 2d, Extended Data Figs. 4b, 5b, 6a; *n* = 4–7). For quantitative analyses of MEs and IMAs in the indicated gastrointestinal regions; alveoli, longitudinal and patch-terminals in the lung; and varicose endings and IMAs in the heart (Extended Data Figs. 5f, 6l, 7b, m, 8d, j, n), the area covered by each sensory ending type was measured using the Leica Application Suite X software and divided by the total area of each sample to derive the innervation intensity. For IGLEs in the gastrointestinal tract, neuroepithelial body (NEB) endings in the lung and flower-spray endings in the heart, the number of terminal clusters was counted (Extended Data Figs. 5f, 6l). The number of IGLEs was divided by the total area of each sample to derive the innervation intensity. Normalized innervation intensity for gastrointestinal endings (Extended Data Figs. 7b, m, 8d, j, n) was calculated as the innervation intensity of the indicated sensory ending type formed in indicated Cre^tdT^ mice divided by the innervation intensity of the indicated sensory ending type formed in Vglut2^tdT^ mice. Fold changes for various sensory ending types in indicated stomach regions (Extended Data Fig. 7d, j) were calculated as the innervation intensity of the corresponding sensory ending type over the indicated stomach region divided by the innervation intensity of the corresponding sensory ending type over the entire stomach in Vglut2^tdT^ mice (*n* = 4).

#### Annotation of VSN clusters.

Heart VSNs were predominantly distributed in four clusters (Extended Data Fig. 5), with the following DEGs: D1 (*Piezo2*), H4 (*Drd2*) and I3/I4 (*Agtr1a*). In Piezo2^tdT^ mice, most cardiac afferents were varicose surface endings. In Drd2^tdT^ mice, cardiac afferents densely innervated myocardium with both branched and parallel IMAs. *Agtr1a*^+^ VSNs predominantly formed flower-spray endings in the heart and the aortic arch. Our results thus reveal the identity of various VSN cardiac ending types (Extended Data Fig. 5h).

Although five types of lung VSN endings were characterized, only two large groups of primary lung VSN clusters were revealed (Extended Data Fig. 6). A3-VSN fibres labelled in Agtr1a^tdT^, Vglut1^tdT^, Pvalb^tdT^ and P2ry1^tdT^ mice travelled along segmental bronchi and terminated at airway bifurcations wrapping around NEBs. Of note, VSN fibres in Agtr1a^tdT^ and P2ry1^tdT^ mice also formed similar endings wrapping around taste buds in the larynx^17^ and the upper oesophagus (Extended Data Fig. 6h), suggesting that VSN bud endings in different organs are likely to have similar genetic signatures. K1–3/L2-VSNs labelled in Twist2^tdT^ and P2ry1^tdT^ mice formed alveoli endings. The other three VSN lung endings that were not effectively marked by Projection-seq were all on bronchial airways, consistent with our observation that lumen-delivered virus did not effectively cover bronchial walls (Extended Data Fig. 1i). *Npy2r*^+^ afferents formed dense longitudinal endings wrapping around the bronchioles; both *Npy2r*^+^ and *P2ry1*^+^ VSNs formed patchy endings on segmental bronchi or around bronchial bifurcations; *Piezo2*^+^ pulmonary afferents mainly formed two ending types: (1) bud ending wrapping around NEBs (Extended Data Fig. 6g) as *Piezo2* is partially in A3-VSNs; and (2) near the bronchial bifurcation with branch endings (Fig. 3e, left), largely from the C3 cluster (1.5% of lung-UPB-marked VSNs). Annotations for lung VSN types are summarized in Extended Data Fig. 6m).

Most stomach VSNs were distributed in nine clusters (Extended Data Fig. 7c). We divided the stomach into five regions based on their proximation to other UPB-targeted organs (Extended Data Fig. 7a, b) and took advantage of dual-UPB labelled VSNs that innervate regions close to both organs to decode each region individually (region 4: stomach/ oesophagus-UPB dual-labelled VSNs; region 6: stomach/pancreas-UPB dual-labelled VSNs; region 7: stomach/colon-UPB dual-labelled VSNs; region 8: stomach/duodenum-UPB dual-labelled VSNs). Compared to other stomach regions, pIMAs and cIMAs but no other endings were enriched around the oesophageal sphincter (Extended Data Fig. 7d, top). Accordingly, three clusters (J1, J3 and I1) were enriched in stomach/ oesophagus-UPB dual-labelled VSNs (Extended Data Fig. 7e, f, top). We then identified DEGs (*P2ry1* for J1/J3 and *Calb2* for I1) and examined IMAs around pyloric sphincters in P2ry1^tdT^ and Calb2^tdT^ mice. Most (75.0%) of the *P2ry1*^+^ IMAs around the oesophageal sphincter were pIMAs and all Calb2^+^ IMAs were cIMAs (Extended Data Fig. 7g), suggesting that J1/J3-VSNs form pIMAs and I1-VSNs form cIMAs. In additional to J1- and I1-VSNs, three clusters were enriched in S/D-VSNs with the following DEGs: F1 (*Sst*, *Gpr65*), H2 (*Vip*) and C4 (*Glp1r*, *Piezo2*), whereas all afferent types in Vglut2^tdT^ mice were enriched in region 8 around the pyloric sphincter. Both *Sst*^+^ and *Gpr65*^+^ VSNs formed MEs on the stomach, as reported^12,16^, with *Sst*^+^ endings mainly in the antrum and *Gpr65*^+^ endings more evenly distributed across the stomach, indicating that F1-VSNs form MEs. Both *Glp1r*^+^ and *Piezo2*^+^ VSNs predominantly formed IGLEs, suggesting that C4-VSNs form stomach IGLEs. *Vip*^+^ VSNs exhibited pIMA morphology in region 8, suggesting that H2-VSNs also form pIMAs (Extended Data Fig. 7d–g).

We also used anatomical tracing results to facilitate the annotation of other stomach VSN clusters. We first determined the relative innervation intensity (referred as anatomical fold change or AF_afferent type-stomach region_) for each ending type (ME, pIMA, cIMA and IGLE) in each stomach region (4, 6, 7 and 8), calculated as the innervation intensity of corresponding ending types in various stomach regions normalized by the innervation intensity of the same ending type in the entire stomach in Vglut2^tdT^ mice (Extended Data Fig. 7j). According to anatomical results (Extended Data Figs. 7, 8), F- and G-VSNs formed mucosal endings (MEs); H2-, H4-, J1- and J3-VSNs formed pIMAs; I1-VSNs formed cIMAs; and C4-VSNs formed IGLEs. We then performed sensory ending type simulation for seven VSN clusters (I2, I4, I5, I6, I7, J2 and J4) in the stomach (Extended Data Fig. 7i) using a MATLAB script, assuming that each could form one of the four ending types (ME, pIMA, cIMA and IGLE) independently. Therefore, in total 4^7^ = 16,384 possibilities were tested. For each possibility, the relative innervation intensity derived from Projection-seq data (referred as Projection-seq fold change or SF_afferent type-stomach region_) of each ending type (ME, pIMA, cIMA and IGLE) in each stomach region (4, 6, 7 and 8) was calculated as the percentage of corresponding dual-labelled VSNs forming this ending type normalized to the percentage of stomach-UPB single-labelled VSNs forming this ending type, expressed as Σ(percentage of dual-labelled VSNs for this stomach region in all VSN clusters that form the corresponding ending type)/Σ(percentage of stomach-UPB single-labelled VSNs in all VSN clusters that form the corresponding ending type). We then calculated the total variance between anatomically and Projection-seq-derived innervation intensities in all stomach regions across all ending types, expressed as Σ(*n* = region 4, 6, 7, 8) (SF_ME-*n*_ − AF_ME-*n*_)^2^ + Σ(*n* = region 4, 6, 7, 8) (SF_pIMA-*n*_ − AF_pIMA-*n*_)^2^ + Σ(*n* = region 4, 6, 7, 8) (SF_cIMA-*n*_ − AF_cIMA-*n*_)^2^ + Σ(*n* = region 4, 6, 7, 8) (SF_IGLE-*n*_ − AF_IGLE-*n*_)^2^. The trial with the lowest variance was defined as the best fit. AF_afferent type-stomach region_ and SF_afferent type-stomach region_ for this condition were plotted together (Extended Data Fig. 7j). Simulation indicated that J2, J4 and I7 account for cIMAs, and I2 and I4–I6 for IGLEs. This prediction was further supported by our data and previous findings^16^ that both *Agtr1a*^+^ and *Oxtr*^+^ VSNs, mainly I-VSNs, formed IGLEs close to the stomach antrum (Extended Data Fig. 7k, l).

Five clusters enriched for duodenum VSNs over stomach/duodenum dual-labelled VSNs were characterized with the following DEGs: *Gpr65* (F4), Vip (H3/G2/G5), *Glp1r* (G2/G5), and *Agtr1a* (I5) (Extended Data Fig. 8a). *Gpr65*^+^ and *Vip*^+^ VSNs predominantly formed indistinguishable MEs arborizing intestinal villi in the duodenum (the density of *Vip*^+^ MEs was much lower), suggesting that both F- and G-VSN clusters form MEs. By contrast, *Agtr1a*^+^ neurons representing I5-VSNs primarily formed IGLEs in the duodenum (Extended Data Fig. 8b–d). Similarly, of the five primary colon VSN clusters, *Agtr1a* is a DEG for I-VSNs and *Agtr1a*^+^ VSNs mainly formed IGLEs in the colon (Extended Data Fig. 8e–g). *Trpv1* is highly expressed in both I- and H4- but only partially in F3-VSNs, and the percentage of IGLE, IMA and ME endings formed by *Trpv1*^+^ VSNs correlated well with the percentage of *Trpv1*^+^ VSNs in I, H4 and F3 clusters (Extended Data Fig. 8h–j). Extensive *Trpv1*^+^ IMAs were observed in the oesophagus, and *Trpv1* is highly expressed in J3 among the five primary oesophagus VSN clusters, consistent with the notion that J3 VSNs form IMAs in the stomach. Piezo2^+^ vagal oesophageal afferents—predominantly from C5–8 clusters—formed dense IMAs, suggesting that unlike C4-VSNs (stomach IGLEs), C5–C8 VSNs also form oesophageal IMAs. Probably owing to low infection efficiency, no apparent oesophageal ME or IGLE clusters were revealed. Notably, among all Cre lines examined, afferents in NTS^tdT^ mice preferentially formed oesophageal IGLEs (Extended Data Fig. 8n) and were later used to study the central projections of oesophageal IGLEs. Nevertheless, oesophageal MEs were predominantly formed by *Trpv1*^+^ and *Gpr65*^+^ VSNs, suggesting they might have similar genetic signatures to MEs in other gastrointestinal organs (Extended Data Fig. 8n, p). Annotations for gut VSN types are summarized in Extended Data Fig. 8q.

#### Correlation analysis.

Correlations among various VSN characteristics, including 7 visceral organs (lung, heart, oesophagus, stomach, pancreas, duodenum and colon), 4 tissue layer types (epithelium, specialized epithelial cells, muscle and elastic connective tissue), 6 VSN ending types (epithelial, budding, pIMA, cIMA, plate of puncta and varicose), 11 VSN subpopulations and 4 response patterns (mechanical sustained, mechanical transient, polymodal and chemical), were plotted in Fig. 3f. Correlation indexes between pairs of VSN characteristics (Fig. 3g) were calculated on the basis of the number and pattern of connections between variables in the two characteristics, with two assumptions: (1) correlation is negatively related to the total number of connections: two characteristics are perfectly correlated (correlation index = 1) if every variable of one characteristic is only connected with one variable of the other characteristic (meaning a one-to-one correlation with the minimum number of possible connections), and two characteristics are completely uncorrelated (correlation index = 0) if every variable of one characteristic is connected to every variable of the other characteristic (meaning the maximum number of possible connections); and (2) with the same number of connections, correlation is stronger if the connections are more evenly distributed, meaning a smaller statistical variance of the number of connections among all variables within both characteristics. We first counted the number of variables within each characteristic (variables with no connections were removed from the analysis) and the number of connections between each pair of characteristics. We then calculated the normalized number of connections (*C*), expressed as (number of connections − minimum number of possible connections)/(maximum number of possible connections − minimum number of possible connections), for each pair of characteristics. We next calculated the statistical variance of connections per variable for both characteristics (v1 and v2). As both variances contribute to the correlation equally, the correlation index was finally calculated as (1 − C)/((v1 + 1) × (v2 + 1)).

### Projection-seq-guided retrograde tracing in the brainstem

Mouse line and organ combinations were selected on the basis of Projection-seq anterograde tracing results: lung alveoli ending (Twist2-ires-Cre, lung), lung NEB ending (Agtr1a-Cre, lung), oesophageal IGLE (Nts-Cre, oesophagus), oesophageal IMA (Piezo2-GFP-ires-Cre, oesophagus), heart IMA (Drd2-Cre, heart), stomach IGLE (Glp1r-ires-Cre, stomach), stomach mucosal ending (Gpr65-ires-Cre, stomach), stomach IMA (Vip-ires-Cre, stomach), duodenal IGLE (Agtr1a-Cre, duodenum), duodenal mucosal ending (Gpr65-ires-Cre, duodenum) and colon IGLE (Agtr1a-Cre, colon). Organ injection of AAVrgs is described in ‘AAV infection of visceral organs’. Brain processing and imaging is described in ‘Histology and immunochemistry’.

### Quantitative analyses of vagal central projections in the brainstem.

Area innervated by vagal afferents retrogradely labelled from various visceral organs at different Bregma levels (−7.20, −7.32, −7.48, −7.56, −7.76, −7.92, −8.0 mm) were measured using Fiji (ImageJ) (Extended Data Fig. 13c). The percentage innervation of indicated brainstem sub-nucleus at certain Bregma level (Fig. 4c, Extended Data Fig. 13d, e) was calculated with the following steps: (1) the fluorescence intensity in each sub-nucleus (FI_Bregma-subnucleus_) was calculated as average fluorescence in the sub-nucleus minus background fluorescence measured in a region in the sub-nucleus with no fluorescence-labelled vagal fibres; (2) the area of each sub-nucleus (A_Bregma-subnucleus_) was measured; (3) the total fluorescence (TF_Bregma_) was calculated as Σ(Bregma, subnucleus) (FI_Bregma-subnucleus_ × A_Bregma-subnucleus_); and (4) the percentage innervation (PI) of a sub-nucleus at a certain Bregma was calculated as FI_Bregma-subnucleus_ × A_Bregma-subnucleus_/TF × 100. The correlation variance between VSNs labelled from various visceral organs (Extended Data Fig. 13f) was calculated as Σ(all subnuclei, all Bregma level) (PI_pathway 1_ − PI_pathway 2_)^2^ and a phylogenetic tree was generated based on the correlation variance matrix using the seqlinkage function in MATLAB.

### Statistics and reproducibility

All statistical analyses were performed using GraphPad Prism 8. All data are reported as mean ± s.e.m. unless specifically mentioned. Significance for pair comparisons was determined and *P* values were reported using a two-tailed Student’s t test. Significance for multiple comparisons was first determined using a one-way ANOVA and adjusted *P* value was then reported using Tukey’s multiple comparisons test.

The sample sizes represent the number of mice used for experiments and data analysis, which is determined according to the consistence of the results. No significant inter-individual variability was observed in our results, suggesting that the sample sizes were sufficient to demonstrate the findings. The precise number of mice is reported in the figure legends and Methods. The study did not report contrasts between treatment and control groups, so randomization was not applicable. For sequencing and tracing experiments, there were no treatment and control groups, and no hypothesis was tested regarding molecular identities. Thus, blinding was not applicable.

Representative images and experiments were repeated independently in multiple mice with similar results: Fig. 2d, Plate of puncta: stomach (*n* = 4), heart (*n* = 3); varicose: heart (*n* = 5), aorta (*n* = 6); pIMA: colon (*n* = 3), heart (*n* = 6); cIMA: stomach (*n* = 4), oesophagus (*n* = 3); epithelial: stomach (*n* = 4), lung (*n* = 5); bud: lung (*n* = 5), oesophagus (*n* = 3); Extended Data Fig. 1a, *n* = 3; Extended Data Fig. 1f, *n* = 2; Extended Data Fig. 1h, i, *n* = 3; Extended Data Fig. 1k–q, *n* = 3; Extended Data Fig. 1r, *n* = 2; Extended Data Fig. 1s, t, *n* = 3; Extended Data Fig. 1v, *n* = 2; Extended Data Fig. 1w, *n* = 4; Extended Data Fig. 2a, *n* = 5; Extended Data Fig. 2b, *n* = 2; Extended Data Fig. 2k, l, *n* = 4; Extended Data Fig. 2p, *n* = 4; Extended Data Fig. 3c, *n* = 4; Extended Data Fig. 4b, oesophagus (*n* = 3), stomach (*n* = 4), duodenum (*n* = 3), colon (*n* = 3), aorta (*n* = 6); Extended Data Fig. 4e, *n* = 3; Extended Data Fig. 5a, *n* = 4; Extended Data Fig. 5g, *Piezo2* (*n* = 9), *Drd2* (*n* = 6), *Agtr1a* (*n* = 3), *Npr2r* (*n* = 2); Extended Data Fig. 6e, *n* = 4; Extended Data Fig. 6g–k, *Npy2r* (*n* = 3), *Trpv1* (*n* = 5), *P2ry1* (*n* = 6), *Agtr1a* (*n* = 4), *Piezo2* (*n* = 9), *Vglut1* (*n* = 4), *Pvalb* (*n* = 3), *Twist2* (*n* = 3); Extended Data Fig. 7g, l, *P2ry1* (*n* = 4), *Calb2* (*n* = 3), *Glp1r* (*n* = 5), *Vip* (*n* = 5), *Gpr65* (*n* = 7), *Sst* (*n* = 5), *Agtr1a* (*n* = 12); Extended Data Fig. 8c, *Glp1r* (*n* = 3), *Agtr1a* (*n* = 4); Extended Data Fig. 8g, *Trpv1* (*n* = 4), *Agtr1a* (*n* = 3); Extended Data Fig. 8o, p, *Nts* (*n* = 5), *Trpv1* (*n* = 3), *Agtr1a* (*n* = 4), *Gpr65* (*n* = 3); Extended Data Fig. 9a, e, f, *n* = 5; Extended Data Fig. 10a, *n* = 5; Extended Data Fig. 13a, lung (*n* = 3), heart (*n* = 3), oesophagus (*n* = 2), stomach (*n* = 3), duodenum (*n* = 3), colon (*n* = 3), pancreas (*n* = 3); Extended Data Fig. 14, *n* = 3 for all groups.

### Reporting summary

Further information on research design is available in the Nature Research Reporting Summary linked to this paper.

### Data availability

The scRNA-seq and Projection-seq data that were produced for this study are available in the Gene Expression Omnibus (GEO) under the accession number GSE192987. The publicly available genome reference mm10 (refdata-cellranger-mm10–3.0.0, ensemble 93) from 10X Genomics was used for scRNA-seq analysis and the generation of Projection-seq genome reference. The AnimalTFDB 3.0 mouse database (http://bioinfo.life.hust.edu.cn/AnimalTFDB/#!/) was used for transcription factor analysis. The Gene Ontology Resource database (http://geneontology.org, release date 1 July 2021) was used for GO pathway analysis. Additional data related to this paper may be requested from the authors. Source data are provided with this paper.

### Code availability

Code for Seurat, Slingshot, tradeSeq and CellPhoneDB is available at https://github.com. Additional code is available upon request.

## Extended Data

## Supplementary Material

Supplemental Table 1

Supplemental Figure 1

Supplemental Information Guide
