# Supplementary material for: A multidimensional coding architecture of the vagal interoceptive system
Source: Nature. Author manuscript; Available in PMC 2022 May 2. (PMC8967724; doi:10.1038/s41586-022-04515-5)
Supplement: Supplemental Figure 1 [file NIHMS1791700-supplement-Supplemental_Figure_1.pdf]

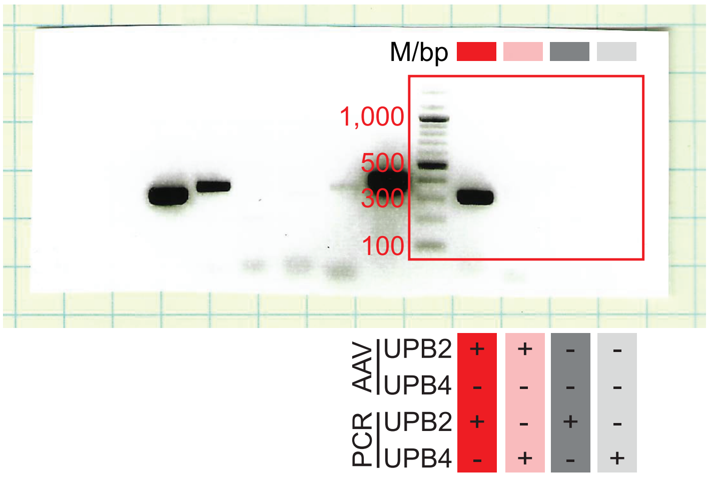

Supplementary Fig. 1. Original source image for electrophoresis, related to Extended Data Fig. 2b. Gel ladder is provided. Red box indicates how the gel was cropped for the final figure.
