# Supplementary material for: A multidimensional coding architecture of the vagal interoceptive system
Source: Nature. Author manuscript; Available in PMC 2022 May 2. (PMC8967724; doi:10.1038/s41586-022-04515-5)
Supplement: Supplemental Information Guide [file NIHMS1791700-supplement-Supplemental_Information_Guide.docx]

Supplementary Fig. 1. Original source image for electrophoresis, related to Extended Data Fig. 2b. Red box indicates how the gel was cropped for the final figure. The file is in PDF format.

Supplementary Table. Detailed information for RNAscope probes. The file is in Excel format.
