# Supplementary material for: A multidimensional coding architecture of the vagal interoceptive system
Source: Nature. 2022 Mar 16;603(7903):878–84. doi: 10.1038/s41586-022-04515-5 (PMC8967724; doi:10.1038/s41586-022-04515-5)
Supplement: Supplementary file 1 [file 41586_2022_4515_MOESM1_ESM.pdf]

nature portfolio

https://doi.org/10.1038/s41586-022-04515-5

Supplementary information

A multidimensional coding architecture of the vagal interoceptive system

In the format provided by the authors and unedited

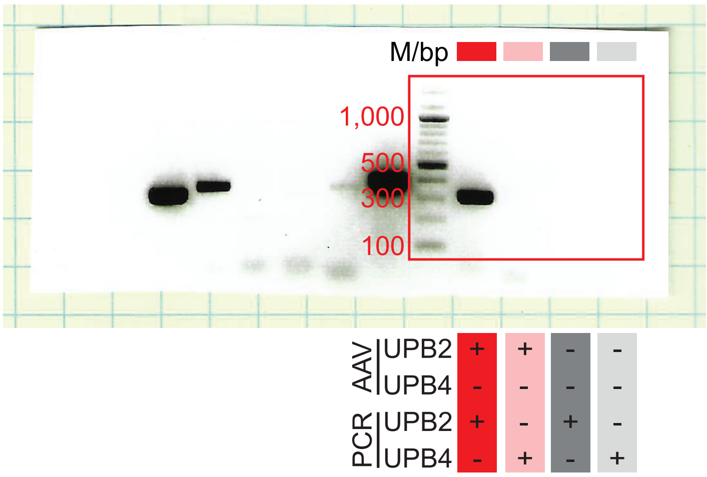

Supplementary Fig. 1. Original source image for electrophoresis, related to Extended Data Fig. 2b. Gel ladder is provided. Red box indicates how the gel was cropped for the final figure.
