# Supplementary material for: A multidimensional coding architecture of the vagal interoceptive system
Source: Nature. 2022 Mar 16;603(7903):878–84. doi: 10.1038/s41586-022-04515-5 (PMC8967724; doi:10.1038/s41586-022-04515-5)
Supplement: Supplementary file 2 [file 41586_2022_4515_MOESM2_ESM.pdf]

nature research

Corresponding author(s): Rui Chang

Last updated by author(s): Jan 10, 2022

Reporting Summary

Nature Research wishes to improve the reproducibility of the work that we publish. This form provides structure for consistency and transparency in reporting. For further information on Nature Research policies, see our Editorial Policies and the Editorial Policy Checklist.

Statistics

For all statistical analyses, confirm that the following items are present in the figure legend, table legend, main text, or Methods section.

n/a	Confirmed	
☐	☒	The exact sample size (*n*) for each experimental group/condition, given as a discrete number and unit of measurement
☐	☒	A statement on whether measurements were taken from distinct samples or whether the same sample was measured repeatedly
☐	☒	The statistical test(s) used AND whether they are one- or two-sided<br>*Only common tests should be described solely by name; describe more complex techniques in the Methods section.*
☒	☐	A description of all covariates tested
☐	☒	A description of any assumptions or corrections, such as tests of normality and adjustment for multiple comparisons
☐	☒	A full description of the statistical parameters including central tendency (e.g. means) or other basic estimates (e.g. regression coefficient) AND variation (e.g. standard deviation) or associated estimates of uncertainty (e.g. confidence intervals)
☐	☒	For null hypothesis testing, the test statistic (e.g. *F*, *t*, *r*) with confidence intervals, effect sizes, degrees of freedom and *P* value noted<br>*Give P values as exact values whenever suitable.*
☒	☐	For Bayesian analysis, information on the choice of priors and Markov chain Monte Carlo settings
☒	☐	For hierarchical and complex designs, identification of the appropriate level for tests and full reporting of outcomes
☒	☐	Estimates of effect sizes (e.g. Cohen's *d*, Pearson's *r*), indicating how they were calculated

Our web collection on statistics for biologists contains articles on many of the points above.

Software and code

Policy information about availability of computer code

Data collection	Sequencing was performed using an Illumina NovaSeq S4 sequencer. Transcriptomic data were aligned to the mm10 mouse genome reference (control scRNA-seq) or a custom mouse genome reference with additional sequence information of UPBs (Projection-seq) using the Cell Ranger software version 3.0.2 (10x Genomics). Confocal and two photon images were collected with Leica Application Suite X (Leica).
Data analysis	ImageJ (Fiji) version 2.0.0-rc-67/1.52d (NIH); Leica Application Suite X (Leica); Prism 8 (GraphPad); Rstudio 2021.09.0 Build 351(Rstudio); Cell Ranger version 3.0.2 (10x Genomics); Ingenuity Pathway Analysis (Qiagen); Matlab 2019a (Matlab); Seurat v3.1 (https://satijalab.org/seurat); CellPhoneDB (https://github.com/Teichlab/cellphonedb, v2.0.0); 3D UMAP Plotting v1.2R (https://github.com/Dragonmasterx87/Interactive-3D-Plotting-in-Seurat-3.0.0); Gene Ontology Resource GO Enrichment Analysis tool (http://geneontology.org, release date 2021-07-01); pheatmap r package version 1.0.12 (https://cran.r-project.org/web/packages/pheatmap/index.html); UMAP (https://github.com/lmcinnes/umap); slingshot (https://github.com/kstreet13/slingshot, v1.6.1); tradeSeq (https://github.com/statOmics/tradeSeq, v1.2.01).

For manuscripts utilizing custom algorithms or software that are central to the research but not yet described in published literature, software must be made available to editors and reviewers. We strongly encourage code deposition in a community repository (e.g. GitHub). See the Nature Research guidelines for submitting code & software for further information.

Data

Policy information about availability of data

All manuscripts must include a data availability statement. This statement should provide the following information, where applicable:

- Accession codes, unique identifiers, or web links for publicly available datasets
- A list of figures that have associated raw data
- A description of any restrictions on data availability

The scRNA-seq and Projection-seq data generated for the current study are available in the Gene Expression Omnibus (GEO) under the accession number GSE192987. Publicly available genome reference mm10 (refdata-cellranger-mm10-3.0.0, ensemble 93) from 10x Genomics was used for scRNA-seq analysis and the generation of Projection-seq genome reference. AnimalTFDB 3.0 mouse database (http://bioinfo.life.hust.edu.cn/AnimalTFDB/#!/) was used for transcription factor analysis. Gene Ontology Resource database (http://geneontology.org, release date 2021-07-01) was used for GO pathway analysis. Additional data related to this paper may be requested from the authors.

Field-specific reporting

Please select the one below that is the best fit for your research. If you are not sure, read the appropriate sections before making your selection.

☒ Life sciences ☐ Behavioural & social sciences ☐ Ecological, evolutionary & environmental sciences

For a reference copy of the document with all sections, see nature.com/documents/nr-reporting-summary-flat.pdf

Life sciences study design

All studies must disclose on these points even when the disclosure is negative.

Sample size	No sample size calculation was performed. For sequencing experiments, sample size was not predetermined. Previous publications (Kupari et al., 2019, Cell Reports; Bai et al.,2019, Cell; Prescott et al., 2020, Cell) and own experience were used to determine approximate number of cells and animals needed for experiments. In our study, we harvested the vagal ganglia from 40 age and gender-matched wildtype mice (10 mice per sample) for control scRNA-sequencing, and 30 age and gender-matched wildtype mice (divided to 4 samples) for Projection-seq. In total, 56,575 cells were sequenced (31,182 control scRNA-seq samples: 7,842, 7,580, 7,939, 7,821; 25,393 Projection-seq samples: 6,403, 6, 381, 5,803, 6,806). All the neuronal subpopulation size in UMAP are over 500 cells, which is with enough depth and beyond the typical standard in the field. For other experiments, the sample sizes represent the number of animals used for experiments and data analysis, which is determined based on the consistence of the results. No significant inter-individual variability was observed in our results. We thus reasoned that the sample sizes were sufficient to demonstrate the findings. The precise number of animals is reported in the figure legends and methods.
Data exclusions	Standard quality control (QC) metrics (number of genes per cell > 500, number of genes per cell < 8000, percentage of mitochondria genes < 10%) were used to filter low-quality cells in scRNA sequencing. These QC metrics were within the range of pre-established criteria in the field: mitochondria gene cut-off between 5% (recommeded by the Seurat package) and 25% (Kupari et al., 2019, Cell Reports, Prescott et al., 2020, Cell); number of genes per cell cut-off between 200-2500 (recommended by the Seurat package for PBMCs) and 2000-10000 (Kupari et al., 2019, Cell Reports). Furthermore, subpopulation E-VSNs selectively express markers for damaged sensory neurons, such as Sprr1a and Ecel1 (Bonilla et al., 2002, J Neurosci; Bai et al., 2019, Cell; Kiryu-Seo et al., 2000, PNAS) and were removed from further analysis. The exclusion criteria were pre-established (Bai et al., 2019, Cell; Prescott et al., 2020, Cell) and are described in the Methods section.
Replication	All experimental findings were reliably reproduced in all experiments. For sequencing experiments, biological replicates and technical quadruplicate were performed. For vCatFISH experiment, biological replicates and independent mice were used. For other experiments, biological and technical replicates were carried out using independent animals. The number of replicates was described in the figure legends and Statistics and reproducibility section.
Randomization	No randomization was performed. The study did not report contrasts between treatment and control groups, hence randomization was not applicable.
Blinding	For the vCatFISH analysis, registration for each sample was carried out by at least two persons independently in a blinded fashion. For sequencing and other experiments, there were no treatment and control groups, and no hypothesis was tested regarding molecular identities. Thus, blinding was not applicable.

Reporting for specific materials, systems and methods

We require information from authors about some types of materials, experimental systems and methods used in many studies. Here, indicate whether each material, system or method listed is relevant to your study. If you are not sure if a list item applies to your research, read the appropriate section before selecting a response.

Materials & experimental systems

n/a	Involved in the study	
☐	☒	Antibodies
☒	☐	Eukaryotic cell lines
☒	☐	Palaeontology and archaeology
☐	☒	Animals and other organisms
☒	☐	Human research participants
☒	☐	Clinical data
☒	☐	Dual use research of concern

Methods

n/a	Involved in the study	
☒	☐	ChIP-seq
☒	☐	Flow cytometry
☒	☐	MRI-based neuroimaging

Antibodies

Antibodies used

chicken anti-GFP (Aves labs, #GFP-1020. Lot: GFP879484); rabbit anti-RFP (Rockland, #600-401-379. Lot: 42896); Alexa Fluor 647-conjugated AffiniPure donkey anti-chicken IgY (Jackson ImmunoResearch, #703-605-155. Lot: 143668); Alexa Fluor 594-conjugated AffiniPure donkey anti-rabbit IgG(H+L) (Jackson ImmunoResearch, #711-585-152. Lot: 142754).

Validation

For chicken anti-GFP (RRID:AB_10000240), validated by manufacturer and previous publications (https://www.aveslabs.com/products/anti-green-fluorescent-protein-antibody-gfp);
For rabbit anti-RFP (RRID:AB_2209751), validated by manufacturer and previous publications (https://www.rockland.com/categories/primary-antibodies/rfp-antibody-pre-adsorbed-600-401-379/);
For Alexa Fluor 647-conjugated AffiniPure donkey anti-chicken IgY, validated by manufacturer and previous publications (https://www.jacksonimmuno.com/catalog/products/703-605-155);
For Alexa Fluor 594-conjugated AffiniPure donkey anti-rabbit IgG(H+L), validated by manufacturer and previous publications (https://www.jacksonimmuno.com/catalog/products/711-585-152).

Animals and other organisms

Policy information about studies involving animals; ARRIVE guidelines recommended for reporting animal research

Laboratory animals

Wildtype C57BL/6J (000664), Glp1r-ires-Cre (029283), Gpr65-ires-Cre (029282), Npy2r-ires-Cre (029285), P2ry1-ires-Cre (029284), Agtr1a-Cre (030553), Calb2-ires-Cre (010774), Nts-Cre (017525), Vglut2-ires-Cre (016963), Sst-ires-Cre (013044), Twist2-Cre (008712), Vip-ires-Cre (010908), Piezo2-GFP-ires-Cre (027719), Trpv1-Cre (017769), Pvalb-Cre (017320), Vglut1-ires2-Cre (023527),Chat-ires-Cre (031661), lox-ChR2 (024109), lox-tdTomato (007914), and Snap25-2A-GCaMP6s-D (025111) were from the Jackson Laboratory. Drd2-Cre (032108-UCD) mice were from the Mutant Mouse Resource and Research Center (MMRRC). All mice were gender and age-matched adults (older than 8 weeks) and no differences between sexes were observed. Mice were maintained less than five mice per cage at 24°C and 56% humidity in a 12h light:12h dark cycle and had ad libitum access to standard food and water by Yale Animal Resource Center.

Wild animals

No wild animals were used in this study.

Field-collected samples

No field-collected samples were used in this study

Ethics oversight

All animal husbandry and procedures were performed in compliance with Yale University’s Institutional Animal Care and Use Committee and National Institutes of Health (NIH) guidelines.

Note that full information on the approval of the study protocol must also be provided in the manuscript.
